# Modulations in the host cell proteome by the hantavirus nucleocapsid protein

**DOI:** 10.1371/journal.ppat.1011925

**Published:** 2024-01-08

**Authors:** Austin Royster, Songyang Ren, Saima Ali, Sheema Mir, Mohammad Mir

**Affiliations:** Western University of Health Sciences, Pomona, California, United States of America; University of Pittsburgh, UNITED STATES

## Abstract

Hantaviruses have evolved a unique translation strategy to boost the translation of viral mRNA in infected cells. Hantavirus nucleocapsid protein (NP) binds to the viral mRNA 5’ UTR and the 40S ribosomal subunit via the ribosomal protein S19. NP associated ribosomes are selectively loaded on viral transcripts to boost their translation. Here we demonstrate that NP expression upregulated the steady-state levels of a subset of host cell factors primarily involved in protein processing in the endoplasmic reticulum. Detailed investigation of Valosin-containing protein (VCP/p97), one of the upregulated host factors, in both transfected and virus infected cells revealed that NP with the assistance of VCP mRNA 5’ UTR facilitates the translation of downstream VCP ORF. The VCP mRNA contains a 5’ UTR of 987 nucleotides harboring six unusual start codons upstream of the correct start codon for VCP which is located at 988^th^ position from the 5’ cap. *In vitro* translation of a GFP reporter transcript harboring the VCP mRNA 5’ UTR generated both GFP and a short polypeptide of ~14 KDa by translation initiation from start codon located in the 5’ UTR at 542^nd^ position from the 5’ cap. The translation initiation from 542^nd^ AUG in the UTR sequence was confirmed in cells using a dual reporter construct expressing mCherry and GFP. The synthesis of 14KDa polypeptide dramatically inhibited the translation of the ORF from the downstream correct start codon at 988^th^ position from the 5’ cap. We report that purified NP binds to the VCP mRNA 5’ UTR with high affinity and NP binding site is located close to the 542^nd^AUG. NP binding shuts down the translation of 14KDa polypeptide which then facilitates the translation initiation at the correct AUG codon. Knockdown of VCP generated lower levels of poorly infectious hantavirus particle in the cellular cytoplasm whose egress was dramatically inhibited in human umbilical vein endothelial cells. We demonstrated that VCP binds to the hantavirus glycoprotein Gn before its incorporation into assembled virions and facilitates viral spread to neighboring cells during infection. Our results suggest that ribosome engagement at the 542^nd^ AUG codon in the 5’ UTR likely regulates the endogenous steady state levels of VCP in cells. Hantaviruses interrupt this regulatory mechanism to enhance the steady state levels of VCP in virus infected cells. This augmentation facilitates virus replication, supports the transmission of the virus to adjacent cells, and promotes the release of infectious virus particles from the host cell.

## Author summary

Valosin-containing protein (VCP), a crucial component in the regulation of protein quality control system, governs diverse cellular processes, including Golgi-ER membrane fusion and Golgi assembly. VCP is translationally upregulated in hantavirus infected cells. Translation initiation from an unusual start codon in the 5’ UTR of the VCP mRNA generates a short polypeptide of ~14KDa in an in vitro translation system. Translation of GFP reporter mRNA, harboring the VCP mRNA 5’ UTR, revealed that synthesis of 14KDa polypeptide negatively impacts the translation of the GFP reporter. We propose that synthesis of 14KDa polypeptide might be a host mechanism to regulate the steady state levels of VCP in cells by controlling the ribosome engagement at the correct start codon for VCP. Our results demonstrate that hantavirus encoded NP binds to the 5’ UTR of VCP mRNA with high affinity. NP shuts down the translation of 14KDa polypeptide in the reporter transcript, which boosts the reporter translation both in cells and in *in vitro* translation system. It is likely that hantavirus use the same strategy to translationally upregulate the VCP during infection. Using gene knockdown and chemical inhibition approaches, we demonstrate that VCP plays a vital role at multiple stages of hantavirus replication and is required for the egress of hantavirus particles from infected cells, a critical event for viral dissemination to neighboring cells.

## Introduction

Hantaviruses are rodent borne zoonotic viruses in the *Hantaviridae* family of the *Bunyavirales* order. Their genome is composed of three negative sense RNA segments S, M and L, which encode nucleocapsid protein (NP), glycoprotein precursor (GPC) and RNA dependent RNA polymerase (RdRp), respectively [1]. The GPC is post-translationally cleaved into two glycoproteins Gn and Gc. Humans are infected by the inhalation of aerosolized excreta from virus infected rodent hosts [2]. Recently human to human transmission has been reported with the Andes hantavirus species in South America [3]. Hantavirus induced hemorrhagic fever with renal syndrome (HFRS) and hantavirus cardiopulmonary syndrome (HCPS), have mortality rates of 15% and 40%, respectively [4]. Although 150,000 to 200,000 cases of hantavirus infection are annually reported worldwide, there is no antiviral therapeutic or FDA approved vaccine for this viral infection.

The interaction between viral and host factors determines the outcome of a viral disease by defining the tissue and organ tropism in the infected host. Viruses have evolved strategies to upregulate the expression of proviral host factors during the course of infection [5]. Recently, comparative CRISPR screens have been conducted for the identification of proviral host factors as potential therapeutic targets for SARS-CoV-2 [6]. To this end we previously reported that hantaviruses have evolved a unique strategy to boost the translation of viral mRNA in virus infected cells [7–10]. We demonstrated that NP specifically binds to both the viral mRNA 5’ untranslated region (5’UTR) and the ribosomal protein S19 (RPS19), a structural component of the 40S ribosomal subunit. The simultaneous binding to both the viral mRNA 5’ UTR and RPS19 selectively engages the NP associated ribosomes on viral transcripts without the requirement of canonical eIF4F cap binding complex. This selective ribosome engagement on viral transcripts boosts their translation. Based on these observations we asked whether NP can also bind to the 5’ UTRs of host cell mRNAs and preferentially facilitate their translation similar to hantavirus mRNA. In this manuscript we demonstrate that a subset of host cell transcripts are efficiently translated in Human umbilical vein endothelial cells (HUVECs) expressing hantavirus NP. While most of these upregulated host factors are related to protein processing in the endoplasmic reticulum, further studies were focused on Valosin-containing protein (VCP/p97/CDC48), one of the upregulated host factors, to provide the mechanistic insight.

We demonstrated that NP boosts the translation of VCP, a member of AAA+ ATPase family, with the assistance of VCP mRNA 5’ UTR. Our results show that preferential translation of VCP by NP-mediated translation strategy is required for the egress of hantavirus particles in infected HUVECs. In addition, VCP likely plays a role in other steps of hantavirus replication cycle. VCP is the widely expressed cytoplasmic proteins in mammalian tissues and is mainly localized in the endoplasmic reticulum (ER). VCP is also known as the transitional endoplasmic reticulum ATPase (TER ATPase) [11,12]. X-ray crystal structure of VCP [13] revealed an N-terminal domain, two middle ATPase domains D1 and D2 [14], and a C-terminal domain in the VCP structure [11,15]. The N-terminal domain is involved in substrate recognition and interaction with adaptors and co-factors. The C-terminal domain binds to a small subset of co-factors and adaptors cooperating with D2 activity. VCP assembles into a functional homohexamer carrying out diverse cellular functions. It plays roles in protein degradation [16], refolding, recycling and relocation, protein trafficking through Golgi [17], autophagy, lipid droplet biogenesis, apoptosis, cell cycle regulation, Golgi-ER membrane fusion and Golgi assembly [11]. The role of differential VCP expression in the biology of different cancers, cancer therapeutic resistance and patient outcomes have been well studied [11,18]. To our knowledge this is the first report demonstrating the role of VCP in viral exit from the host cell, a critical step in the virus replication and propagation to neighboring cells. The results reported here suggest that VCP could be a potential therapeutic target for anti-hantavirus drug discovery.

## Results

### Alterations in human umbilical vein endothelial cell (HUVEC) proteome due to co-expression of hantavirus nucleocapsid protein

**Fig 1:**
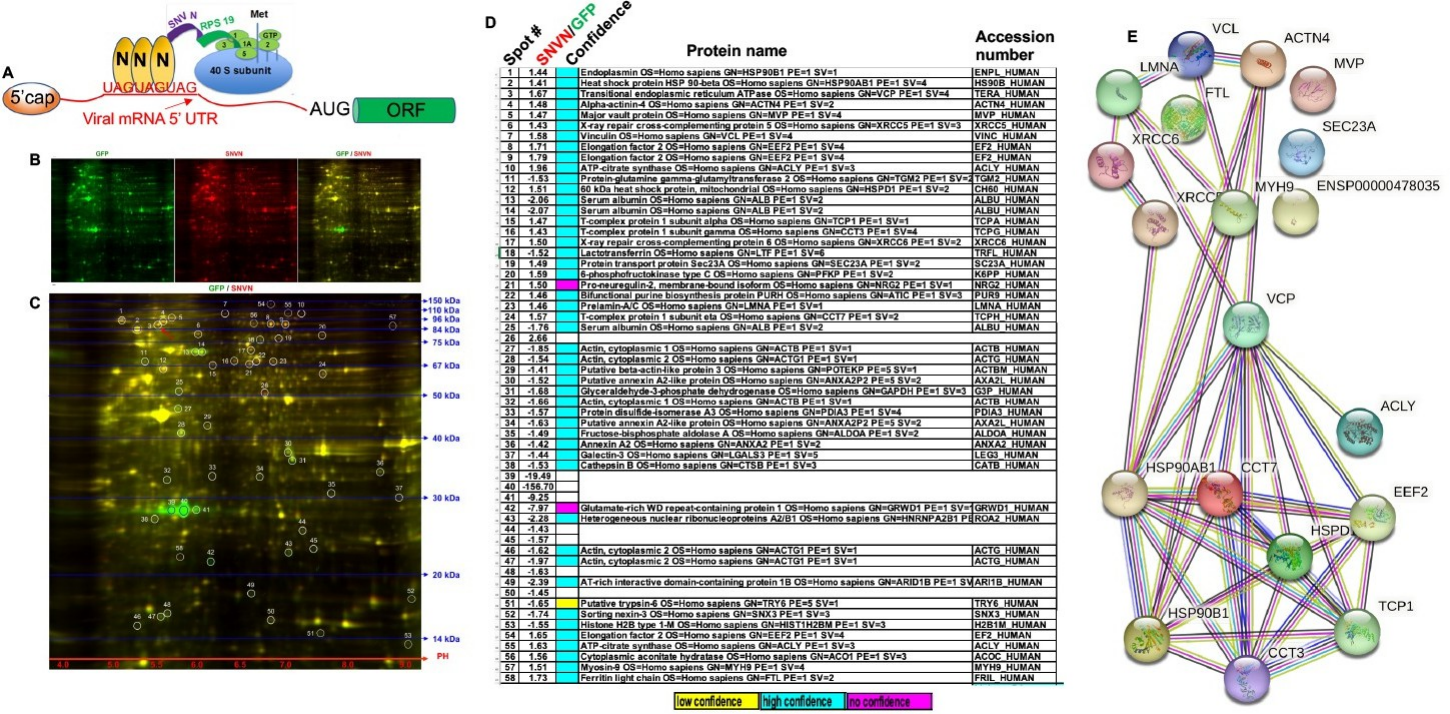
Alterations in the host proteome by the co-expression of hantavirus NP. **(A)**. Model showing the NP-mediated translation initiation mechanism. The nucleocapsid protein is shown by letter “N”. **(B)**. 2D-DIGE analysis of HUVEC lysates expressing either GFP or Sin Nombre virus nucleocapsid protein (SNVN). The cell lysates from GFP and NP expressing cells were labelled with cy3 (green) and cy5(red), respectively. The overlay of cy3 (green) and cy5 (red) images is shown on the right (GFP/SNVN). **(C)**. Zoomed view of the overlay image showing 54 well resolved encircled protein spots. The protein ladder and PH scale are shown. **(D)**. The cy5 (red) and cy3 (green) signals of each protein spot in panel C was calculated and the ratio of the signals was reported in panel D. The spots were excised from the gel and protein identification was carried out by mass spectrometry (see Materials and methods for details). **(E)**. The string analysis of the host factors whose intrinsic protein levels were higher in NP expressing cells. Nodes represent the proteins, and edges represent the protein-protein associations. The associations are meant to be meaningful, indicating that proteins could jointly contribute to a shared function without necessarily meaning that they are physically binding to each other. The known interaction from curated databases (**—)** and experimentally verified data sets (**—**) are shown. The predicted interaction from gene neighborhood (**—**), gene fusions (**—**), and gene co-occurrences (**—**) are shown. Other interactions based on textmining (**—**), co-expression (**—**) and protein homology (**—**) are shown.

The majority of eukaryotic mRNA translation is m7G cap dependent and is initiated by the formation of eIF4F complex on the mRNA 5’ cap. The eIF4F cap binding complex, also called ribosome landing pad, is composed of three initiation factors eIF4E, eIF4A, [19,20] and eIF4G (33). Viral mRNAs have to compete with host cell transcripts for the same translation apparatus. However, viruses have evolved numerous strategies to avoid such competition and facilitate the efficient translation of viral mRNA in the host cell. We previously reported that hantavirus NP from either new world hantaviruses (Sin Nombre virus, Prospect Hill virus, Andes virus) or old-world hantaviruses (Hantaan virus) lures the host translation machinery for the preferential translation of viral mRNA [7–10,21,22]. We showed that NP binds to the ribosomal protein S19 (RPS19), a structural component of the 40S ribosomal subunit [23]. NP also binds to a highly conserved triplet repeat sequence (UAGUAGUAG) of the viral mRNA 5’ untranslated region (5’UTR) (18, 20). NP associated ribosomes are preferentially loaded on viral mRNA 5’ UTR using an IRES type of mechanism, to boost the translation of downstream open reading frame (ORF) (see the model in Fig 1A). NP mediated translation strategy does not require eIF4F cap binding complex.

**Fig 2:**
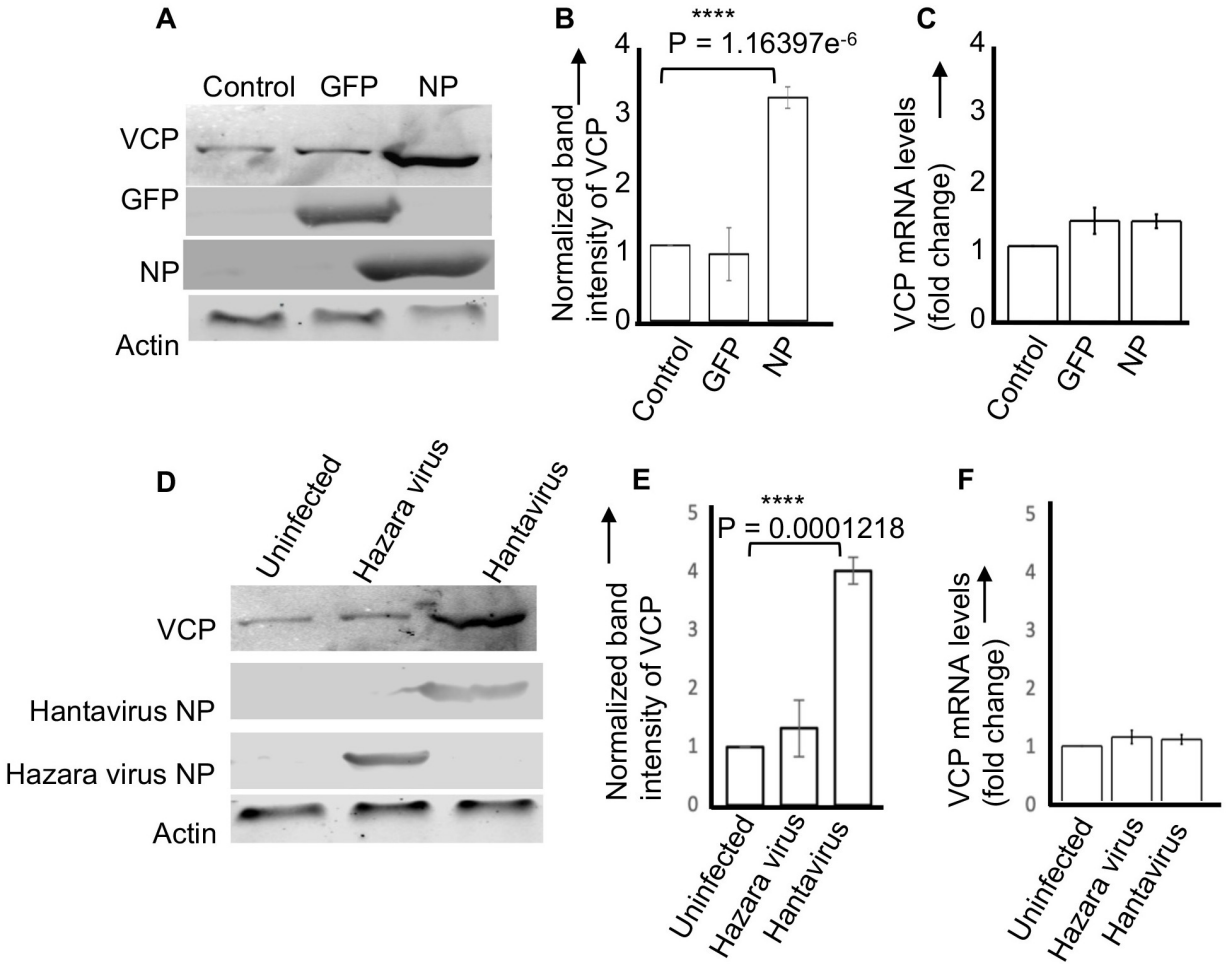
NP facilitates the translation of VCP mRNA. **(A)** HUVECs in six well plates were infected with lentiviruses expressing either GFP or SNV NP or were uninfected (control). Cell lysates were examined by western blot analysis using appropriate antibodies. **(B)** The band intensities of VCP in panel A were quantified and normalized related to control. The normalized intensity values from three independent experiments were averaged and plotted in panel B. **(C)** Quantification of VCP mRNA levels by real time PCR, relative fold changes compared to control are shown. **(D)** A western blot showing the VCP protein levels in HUVEC lysates, generated from either uninfected cells or cells infected with hazara virus or hantavirus (PHV). **(E)** The VCP band intensities in panel D were quantified, normalized related to control, and plotted in panel E, as mentioned in panel B. **(F)** A real time PCR analysis showing the relative VCP mRNA levels in cell lysates from panel D, relative fold changes compared to control are shown. Note: The “P” values were calculated by students *t*-test.

We asked whether NP-mediated translation mechanism can boost the translation of host cell mRNAs using the similar strategy. To test this hypothesis lentiviruses expressing either Sin Nombre virus nuclepcaspid protein (NP) or GFP were generated, as mentioned in Materials and Methods. HUVECs grown in 10 cm dishes were infected with the resulting lentiviruses and cells were lysed 48 hours post-infection in RIPA lysis buffer (Fisher Scientific). As discussed in detail in Materials and Methods section, the cell lysates were examined by 2D-DIGE to determine whether co-expression of NP influenced the intrinsic steady state levels of host proteins in comparison to GFP control. As shown in Fig 1B and 1C, the green (cy3) and red (cy5) signal of fifty-eight well resolved protein spots was quantified and the resulting fold change is shown in Fig 1D. It is evident from Fig 1D, that about 50% of the spots showed higher protein levels in NP expressing cells in comparison to cells expressing GFP. Each of the 58 protein spots were excised from the gel and their identity was determined by mass spectrometry, as discussed in detail in the Materials and Methods section. The mass spectrometric analysis revealed with high confidence the identity of majority of the spots (Fig 2D). The spot 26 (Fig 1C) represents the SNV NP, based on the molecular weight of NP (~53 KDa) and highest Cy5 (red) signal in NP expressing cells. Similarly, the spots 39–41 represent the GFP, consistent with highest cy3(green) signal and molecular weight of GFP (~27 KDa). The differential migration of GFP (spots 39–41) is likely due to post-translational modification of GFP. The proteins with higher intrinsic steady state levels in NP expressing cells were: Endoplasmin, Heat shock protein HSP 90-beta,Transitional endoplasmic reticulum ATPase (VCP), Major vault protein, Alpha-actinin-4, X-ray repair cross-complementing protein 5, Elongation factor 2, ATP-citrate synthase, 60 kDa heat shock protein, Ferritin light chain, Myosin-9, Vinculin, T-complex protein 1 subunit α, T-complex protein 1 subunit χ, X-ray repair cross-complementing protein 6, Protein transport protein Sec23A, Isoform 1 of Pro-neuregulin-2 membrane-bound isoform, Prelamin-A/C and T-complex protein 1 subunit ε.

All these proteins expressed at higher levels in NP expressing cells, were examined by STRING analysis, an online tool, to determine potential functional correlations. The STRING analysis tool predicts potential correlations based on several factors including known interactions verified experimentally or curated from data bases, or predicted interactions based on gene neighborhood, gene fusions or gene co-occurrence, or other evidence such as protein homology or co-expression (Fig 1E). Functional analysis of the identified proteins by KEGG pathway enrichment reveled that majority of these proteins are related to protein processing in the endoplasmic reticulum, and transitional endoplasmic reticulum ATPase (VCP) is linked to most of these predicted functional networks (Fig 1E). To confirm that NP regulates the expression of these host factors at translational level further studies were focused on VCP.

### NP facilitates the translation of VCP mRNA

To further confirm that higher intrinsic steady state levels of VCP in NP expressing cells were due to NP-mediated translation strategy, we monitored the VCP levels in cell lysates by western blot analysis and quantified the VCP mRNA levels by real time PCR. Briefly, HUVECs infected with lentiviruses expressing either SNV NP or GFP were lysed 48 hours post-infection and cell lysates were examined to monitor the VCP protein and mRNA expression levels. As shown in Fig 2A and 2B, the VCP protein levels were significantly higher in NP expressing cells as compared to cells expressing GFP. We didn’t observe any change in the VCP mRNA levels in NP and GFP expressing cells (Fig 2C). Similar results were obtained with NP from either Hantan virus or Prospect Hill virus.

To further confirm these results, HUVECs were infected with either Prospect Hill virus (PHV) or hazara virus (HZV), another member of the order *Bunyavirales*. Cell lysates were examined for VCP mRNA and protein levels. As shown in Fig 2D–2F, VCP protein levels were significantly higher in PHV infected cells (Fig 2D and 2E) without any alteration in mRNA levels (Fig 2F). Taken together, the results from Fig 2 demonstrate that co-expression of hantavirus NP likely facilitated the translation of VCP mRNA, although the post-translational stabilization of VCP protein by the co-expression of NP cannot be ruled out at this point.

### NP with the assistance of VCP mRNA 5’ UTR facilitates the translation of downstream ORF

**Fig 3:**
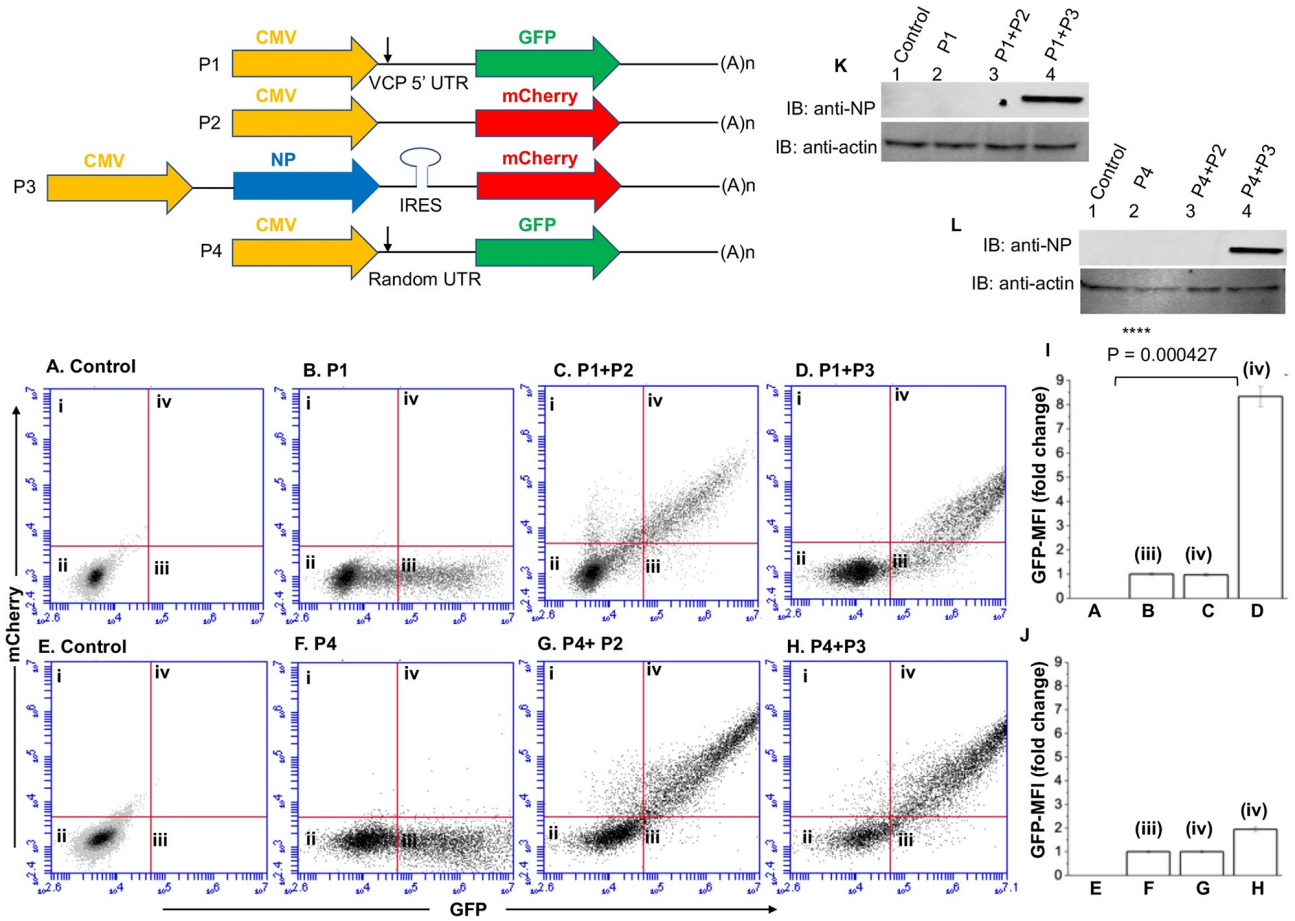
An examination by flow cytometry to further confirm the NP-mediated translation of reporter mRNA. The plasmids (P1, P2, P3 and P4) used in this study are shown at the top. For the construction of these palsmids please see Materials and methods. HUVECs in twenty four well plates were transfected with either Plasmid P1 **(panel B)** or cotransfected with plasmid P1 and P2 **(panel C)** or Plasmid P1 and P3 **(panel D)**. Panel A shows the mock transfected cells. Cells were examined by flow cytometry 24 hours post-transfection. Mean GFP signal from GFP positive cells in quadrant III (panel B) and both GFP and RFP postive cells from quadrant iv (panels C and D) was calculated. The mean GFP fluorescence value from panels A,B,C and D was normalized relative to panel B and plotted in pannel I. Cells in **panels E, F, G and H** were transfected and examined similar to panels A,B,C and D, except the plasmid P1 was replaced by the plasmid P4. The quantified GFP signal was analyzed and plotted in panel **J** as mentioned in panel **I**. Panles **K** and **L** show the western blot analysis of samples from panels A-H, using anti-NP antibody.

Since NP with the assistance of viral mRNA 5’ UTR facilitates the mRNA translation, we asked whether NP can facilitate the translation of VCP mRNA with the assistance of its 5’ UTR. We generated a reporter plasmid P1 expressing GFP mRNA harboring the 5’ UTR of VCP mRNA upstream of the start codon (Fig 3). The plasmid P4 was generated similarly except the 5’ UTR sequence of VCP mRNA was randomized. We generated an NP expression construct (plasmid P3, Fig 3) expressing NP by cap dependent translation and mCherry reporter from Hep C virus IRES. Thus, all mCherry positive cells transfected with plasmid P3 also express NP, which was confirmed by western blot analysis (Fig 3K). The control plasmid P2 expresses mCherry alone. HUVECs were co-transfected with the reporter construct of interest (plasmid P1 or plasmid P4) along with either plasmid P3 or plasmid P2. The co-transfected cells were examined by flow cytometry and GFP signal in cells positive for both GFP and mCherry was quantified and plotted in Fig 3 (panels I and J). It is evident that NP expression, confirmed by western blot analysis (Fig 3K and 3L), selectively favored the translation of GFP reporter mRNA expressed from plasmid P1 in cells expressing both the GFP reporter and NP (Fig 3D and 3I). Interestingly, the randomization of VCP mRNA 5’ UTR sequence abolished the preferential translation of the reporter mRNA by NP-mediated translation strategy (Fig 3H and 3J). These studies clearly demonstrate that NP with the assistance of VCP mRNA 5’ UTR favors the translation of downstream ORF.

### NP shuts down the translation initiation from an upstream start codon in the VCP mRNA 5’ UTR

**Fig 4:**
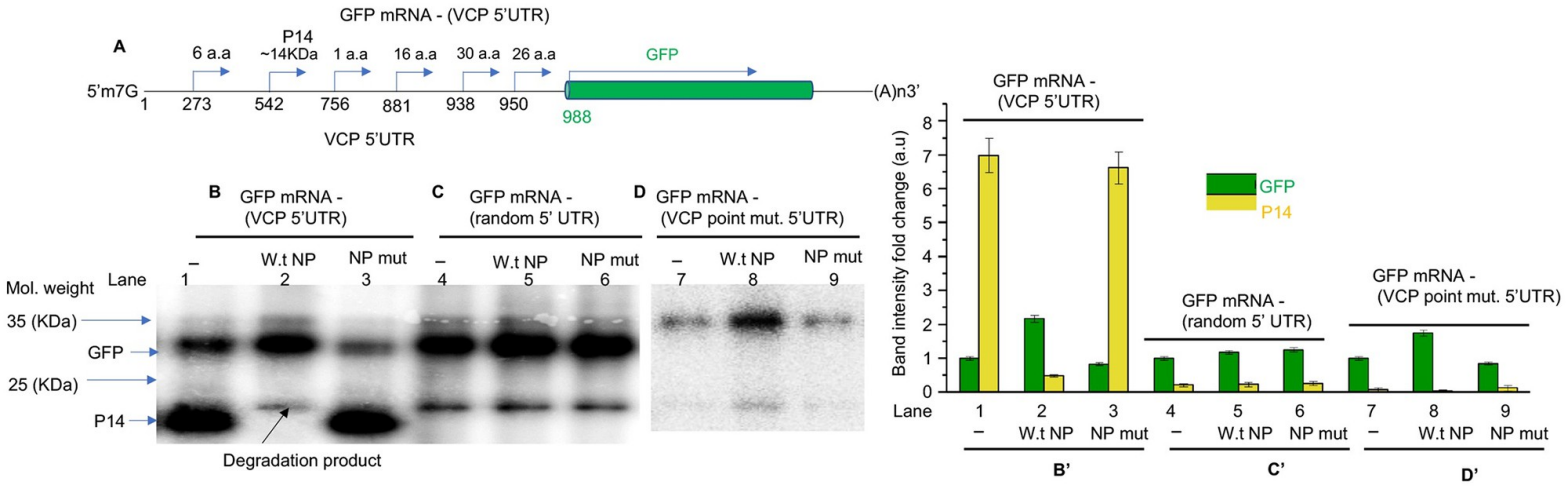
NP shuts down the translation initiation from an upstream start codon in the VCP mRNA 5’ UTR. **(A)** Pictorial representation of the GFP mRNA—(VCP 5’UTR), generated by fusing the VCP mRNA 5’ UTR to the GFP OFR upstream of the start codon. Shown is the location of start codons in the 5’ UTR of VCP mRNA. The three transcripts: GFP mRNA-(VCP5’UTR) **(panel B)**, GFP mRNA-(random 5’ UTR) **(panel C)**, GFP mRNA-(VCP point mut. 5’ UTR) **(panel D)** were translated in rabbit reticulocyte lysates and radiolabeled translation products were separated in SDS-PAGE gel. The intensity of bands corresponding to GFP and P14 in lanes 1,2 and 3 (panel B) were quantified and normalized related to the GFP band intensity in lane 1. The normalized intensity values were plotted in **(panel B’)** on right. Similarly, the intensity of bands corresponding to GFP and P14 in lanes 4, 5 and 6 (panel C) were quantified and normalized related to the GFP band intensity in lane 4. The normalized intensity values were plotted in **(panel C’)** on right. Same strategy was followed to quantify the band intensities in lanes 7, 8 & 9. The intensities were normalized related to the GFP band intensity in lane 7 and plotted in **(panel D’)**

The VCP mRNA harbors a 5’ UTR of 987 nucleotides (ID: NM_001354928.2) that contains six start codons located at positions 273, 542, 7756, 881, 938, 950 (Fig 4A). Translation initiation at these start codons can potentially generate short polypeptides, except the start codon at the position 542 that can generate a polypeptide of ~ 14KDa. The correct start codon for VCP ORF is located at the position 988 that generates VCP of ~97KDa. We used plasmid P1 to synthesize the 5’ capped GFP mRNA harboring the VCP mRNA 5’UTR by *in vitro* T7 transcription reaction, as previously reported [8,10,21,22,24]. The mRNA is referred as GFP mRNA-(VCP5’UTR) (Fig 4A). This mRNA was translated in rabbit reticulocyte in the presence of bacterially expressed and purified wild type NP or NP mutant lacking the RNA binding domain. The translation products were labeled with S^-35^ methionine during synthesis and separated on SDS-PAGE gel. An examination of the gel by phosphorimage analysis showed two prominent translation products corresponding to GFP (~27KDa) and an unknown protein of ~14 KDa referred as P14 (Fig 4B, lane 1). The P14 was likely generated by translation initiation from the start codon at position 542 in the VCP mRNA 5’ UTR. The band intensity was quantified and plotted in Fig 4B’. Interestingly, the addition of purified wild type NP to the translation reaction dramatically inhibited the synthesis of P14 and increased the synthesis of GFP by ~ 2-fold (Fig 4B and 4B’, lane 2). The addition of NP mutant had no impact upon the translation of GFP mRNA-(5’UTR) (Fig 4B, lane 3).

We next used the plasmid P4 to synthesize the GFP mRNA harboring the randomized sequence of VCP mRNA 5’ UTR, referred as GFP mRNA-(random 5’UTR). This mRNA contained a single start codon at the position 988 and lacked all upstream start codons in the 5’ UTR. The mRNA was similarly translated in rabbit reticulocyte lysates in the presence of wild type NP and NP mutant lacking the RNA binding domain. As shown in Fig 4C and 4C’, this mRNA generated GFP and not P14. Its translation was not affected by the addition of wild type or mutant NP. To further confirm these observations, we synthesized the GFP mRNA harboring the VCP mRNA 5’ UTR that contained the point mutation converting the 542^nd^ AUG to AUC. This mRNA referred as GFP mRNA-(VCP point mut. 5’UTR) (Fig 4C) was similarly translated in the presence of wild type and mutant NP. Translation of this mRNA also didn’t generate the 14KDa protein and the translation of GFP was slightly increased by the addition of wild type NP (Fig 4D and 4D’). Taken together, these results clearly demonstrate that translation initiation from the start codon located 542 nucleotides from 5’ cap in the 5’ UTR of VCP mRNA generates an unknow 14KDa protein in an *in vitro* translation system. The addition of purified NP to the translation system shuts down the translation initiation from this start codon that results in an increase in the GFP translation product, probably by efficient ribosome engagement at the downstream start codon for GFP (codon 988). Since we did not notice any other translation products in the gel except P14 and GFP, it is likely that ribosomes do not engage at other start codons located in the VCP mRNA 5’ UTR.

### A bi-cistronic reporter mRNA demonstrates the translation initiation in cells from an upstream AUG located 542 nucleotides from the 5’ cap in the VCP mRNA 5’ UTR

**Fig 5:**
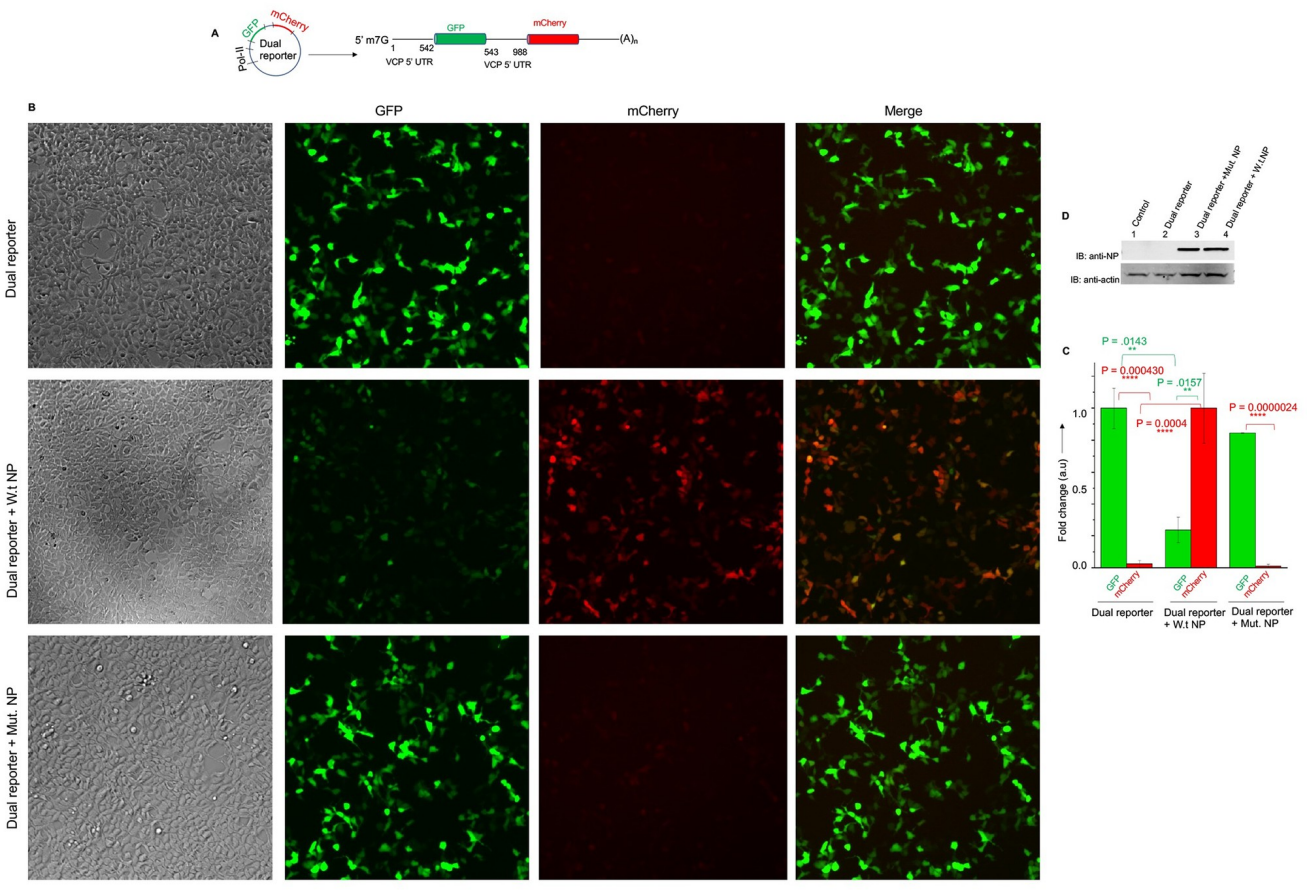
Translation of bi-cistronic mRNA in cells analyzed by fluorescence microscopy. **(A)** The dual reporter construct expressing the bi-cistronic mRNA from the pol-II promoter. **(B)**. Fluorescence microscopy of cells transfected with dual reporter construct along with another construct expressing either wild type NP or NP mutant lacking the RNA binding domain. **(C)** The GFP and mCherry signal in cells from panel B was quantified. The quantified GFP signal was normalized related to control transfected with dual reporter construct alone. The mCherry Signal was normalized related the cells co-transfected with dual reporter construct and wild type NP expression construct. (**D**). Western blot analysis of cell lysates from panel (B) using anti-NP antibody. Control (lane 1) represents the untransfected cells. Note: NP mutant is 5KDa smaller in size compared to W.t NP, this small difference in Molecular weight is not noticeable in the 10% acrylamide gel.

We generated a dual reporter construct (Fig 5A) to further confirm the results from Fig 4 that NP shuts down the translation initiation at the 542^nd^ AUG codon in the 5’ UTR in order to facilitate the translation initiation at correct downstream initiation codon for GFP (988^th^AUG). Briefly, the GFP ORF was incorporated downstream of the AUG codon located at 542^nd^ position in the 5’ UTR of VCP mRNA and the mCherry ORF was incorporated downstream of the correct initiation codon located at 988^th^ position in the 5’ UTR (Fig 5A). The bi-cistronic mRNA expressed from the dual reporter construct will generate GFP signal in cells if the ribosomes loaded at the 5’ cap initiate translation at 542^nd^ start codon. Failure to engage at the 542^nd^ AUG, the ribosomes will likely keep scanning the UTR and engage at the 988^th^AUG to generate the mCherry signal in cells. This is based on observations from Fig 4 that all other AUG codons in the VCP mRNA 5’ UTR are likely weak and do not engage ribosomes. However, the GFP ORF contains five intrinsic AUG codons encoding methionine in the ORF, it is likely that ribosomes failing to engage at the 542^nd^ AUG in the 5’ UTR will engage at AUG codons located in the GPF ORF. To avoid such engagement, we generated a methionine free GFP in which all intrinsic AUG codons in the GFP ORF were mutated. It has been previously reported that mutation of all five intrinsic AUG codons in the GFP ORF that converted five methionine residues to other amino acids (M78I, M88L, M153T, M218A and M233K) generated a weakly fluorescent GFP due to complications in protein folding [25]. However, incorporations of additional twelve mutations (S30R, Y39N, F64L, F99S, N105T, Y145F, N149K, M153T, V163A, I171V, A206V and S208L) created a highly stable and properly folded GFP molecule whose fluorescence quantum yield was significantly higher compared to the wild type GFP molecule [25]. All these mutations were incorporated in the GFP ORF to create mutant GFP devoid of intrinsic methional residues. The DNA segment encoding the bi-cistronic mRNA (Fig 5A) was synthesized and incorporated in the pCDNA 3.1 (+) backbone to create the dual reporter construct.

HUVECs in six well plates were transfected with dual reporter construct along with another plasmid expressing either wild type NP or NP mutant lacking the RNA binding domain. GFP and mCherry signals were recorded in transfected cells 36 hours post transfection by fluorescence microscopy (Fig 5B). The quantified signals were plotted in Fig 5C. It is evident from Fig 5B, that ribosomes preferentially initiated translation at the 542^nd^ AUG, generating a strong GFP signal. The translation initiation at the 988^th^ AUG was significantly weak evident from negligible mCherry signal. However, co-expression of NP significantly inhibited translation initiation at 542^nd^ AUG and promoted the initiation at 988^th^ AUG codon, evident from diminished GFP signal and enhanced mCherry signal (Fig 5B and 5C). The NP mutant had no impact upon the translation of bi-cistronic mRNA. The expression of wild type and mutant NP is shown in Fig 5D.

**Fig 6:**
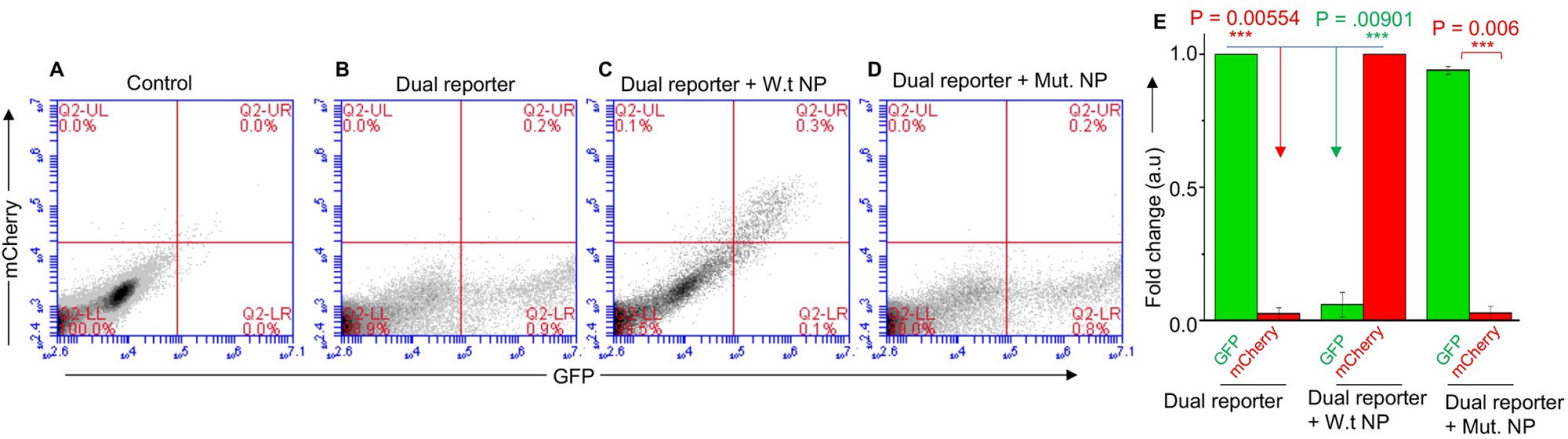
Translation of bi-cistronic mRNA in cells analyzed by flowcytometry. Flow cytometric analysis showing GFP and mCherry expression in HUVECs transfected with dual reporter construct (**B**) along another plasmid expressing either wild type NP **(C)** or mutant NP **(D)**. The mean GFP and mCherry fluorescence signal in panels B, C and D was recorded and normalized as mentioned in Fig 5C. The normalized signal was plotted in panel (**E**). The control is shown in panel **(A)**.

The above transfected cells were also examined by flow cytometry (Fig 6). Consistent with the results from Fig 5, again a strong GFP signal and a week mCherry signal was observed in cells transfected with the dual reported construct alone (Fig 6B and 6E). The GFP signal was significantly decreased and the mCherry signal was significantly increased in cells co-transfected with wild type NP expression construct (Fig 6C and 6E). Again, the co-expression of NP mutant had no impact upon the translation of bi-cistronic report mRNA (Fig 6D and 6E). Taken together, the results from both *in vitro* translation (Fig 4) and cell culture studies (Figs 5 and 6) clear demonstrate that translation is strongly initiated at the 542^nd^ AUG codon located in the VCP mRNA 5’ UTR which negatively impacted the translation of the ORF downstream of 988^th^ AUG codon. Co-expression of NP shuts down the translation initiation at the 542^nd^ AUG which in turn boosts the translation of the ORF located downstream of 988^th^ AUG.

### NP binds to the VCP mRNA 5’ UTR with high affinity

**Fig 7:**
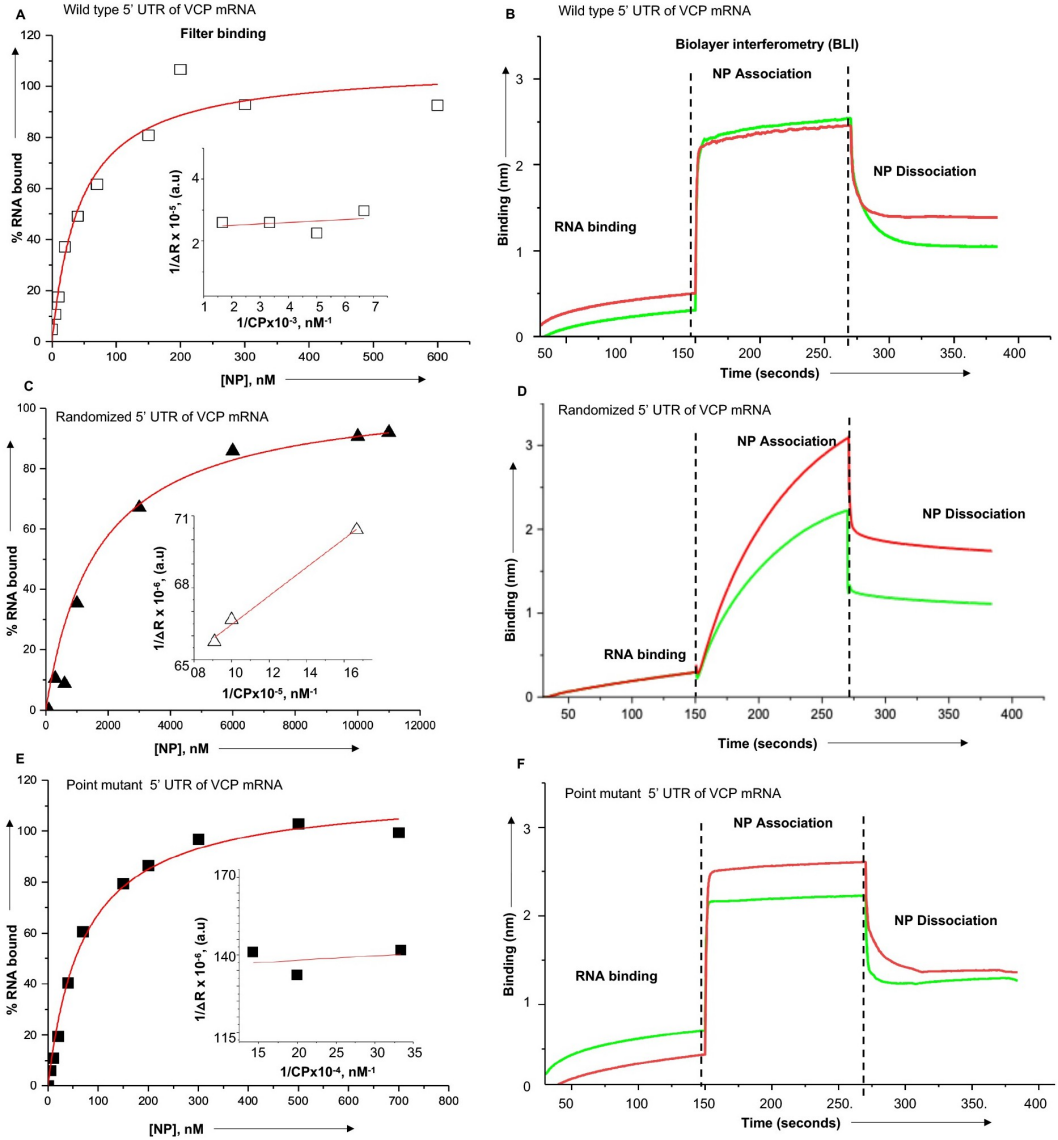
Binding of NP with wild type and mutant sequences of VCP mRNA 5’ UTR using filter binding analysis and biolayer interferometry. Representative binding profiles for the interaction of NP with the wild type sequence of VCP mRNA 5’ UTR (panels **A & B**), randomized sequence of VCP mRNA 5’ UTR (panels **C&D**) and point mutant of VCP mRNA 5’ UTR (**E&F**). Binding profiles generated by filter binding analysis and biolayer interferometry are shown on the left side and right side, respectively. The binding profiles were generated as discussed in materials and methods. The red and blue lines in binding profiles generated by biolayer interferometry (B, D & F) represent the binding analysis carried out at two different concentrations of NP.

We next asked whether shutdown of translation initiation at the 542^nd^ AUG is due to high affinity binding of NP to the VCP mRNA 5’ UTR. As discussed in materials and methods, we used two independent experimental approaches (filter binding analysis and biolayer interferometry) to monitor the binding of bacterially expressed and purified NP with the synthetic wild type 5’ UTR of VCP mRNA, randomized 5’ UTR of VCP mRNA and point mutant 5’ UTR of VCP mRNA harboring a point mutation that converted 542^nd^ AUG to AUC. These UTR sequences were either radiolabeled with P^32^-GTP or biotinylated during synthesis (see materials and methods for details). As shown in Fig 7 (Panels A & E) and Table 1, filter binding analysis revealed that NP bound to the wild type and point mutant 5’UTR with the dissociation constant (K_d_) values of ~47±10, nM and ~71±7, nM, respectively, demonstrating the similar binding affinity. In comparison the binding affinity with the mutant 5’ UTR (Fig 7C and Table 1) was significantly weaker, evident from high K_d_ value ~1.6±0.5 μM. We used biolayer interferometry to further confirm these results (see materials and methods for details). As shown in Fig 7 (panels B & F), NP bound to both the wild type and point mutant of VCP mRNA 5’ UTR with fast on-rate and slower off rate (Table 1), generating the dissociations constant (Kd) values of ~25±13 nM and ~16±4, nM, respectively (Table 1). In comparison NP bound to randomized 5’ UTR with slower on and off rates, generating a dissociation constant of ~2.86±0.2, μM. Thus, it is clear from both the studies that binding affinity of NP with the wild type and point mutant UTR sequences of VCP mRNA 5’ UTR was in nano-molar range when the binding affinity with the randomized UTR was in micro molar range.

**Table 1:** Filter binding and Biolayer interferometry to study the interaction of NP with wild type and mutant sequences of the VCP mRNA 5’ UTR.

Interactors	Filter binding	Biolayer interferometry		
	K_d_ ±SD	K_d_ ±SD	K_ass_(M^-1^S^-1^)	K_dis_(S^-1^)
NP + wild type 5’UTR	47±10, nM	25±13, nM	4.804x10^6^	1.231x10^-1^
NP + Randomized 5’UTR	1.6±0.5, μM	2.86±0.2, μM	8.139x10^3^	2.325x10^-2^
NP + point mutant 5’UTR	71±07, nM	16±4, nM	8.517x10^6^	1.42x10^-1^

Note: K_d_ = K_dis_/K_ass_.

### The nucleotides from 530–542 of the VCP mRNA 5’UTR are required for high affinity binding to NP

**Fig 8:**
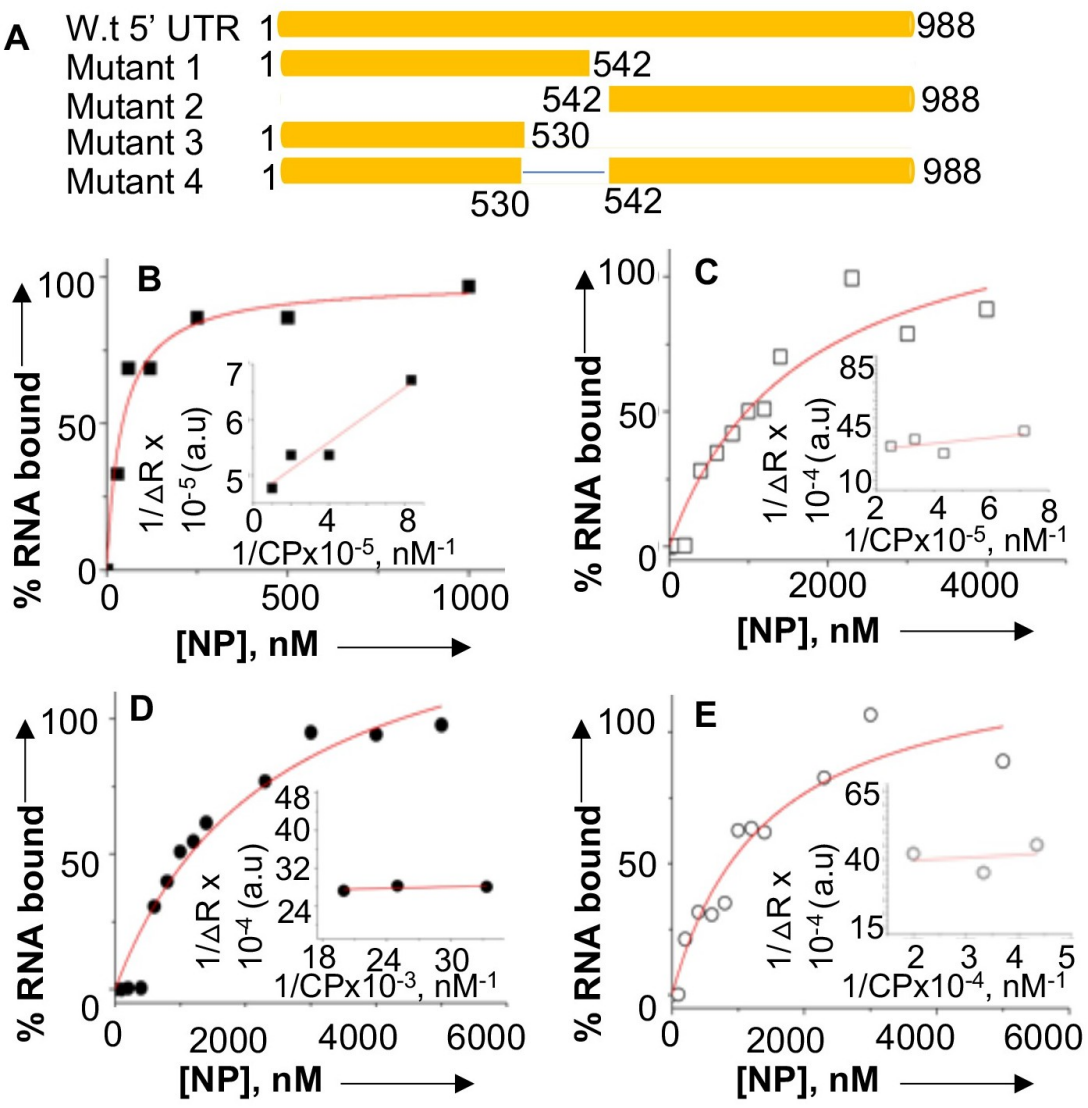
Binding profiles for the interaction of NP with the mutant sequences of VCP mRNA 5’ UTR using filter binding approach. (**A)** Pictorial representation of the VCP mRNA 5’ UTR and its mutants. Shown are the representative binding profiles for the interaction of NP with the mutant 1 (**B**), mutant 2 (**C**), mutant 3 (**D**) and mutant 4 (**E**) in the RNA binding buffer at 80 mM NaCl. The binding profiles were generated using filter binding analysis, as mentioned in materials and methods.

To determine the region of VCP mRNA 5’UTR that plays a role in the high affinity binding, we synthesized and radiolabeled the 542 nucleotides from 5’ end of the UTR corresponding to the region from nucleotides 1–542 (Fig 8A, Mutant 1). Similarly, 446 nucleotides from 3’ end of the UTR corresponding to the region from 542–988 nucleotides (Fig 8A, Mutant 2) were synthesized. Using filter binding analysis, we examined the binding of both the mutants with NP at increasing concentrations of NaCl. As shown in the representative binding profiles (Fig 8) and Table 2, NP bound to the mutant 1 with high affinity similar to wild type UTR (K_d_ ~42.2±10.5 nM). We did not observe any noticeable change in the binding affinity at increasing salt concentrations (Table 2), demonstrating the specificity for binding. In comparison, the binding affinity of NP to the mutant 2 was significantly weaker, which was further impacted at higher salt concentrations (Table 2). To gauge further insight into the binding site on 5’ UTR, we generated another mutant (Fig 8A, Mutant 3) which is similar to mutant 1 except it lacks the short region from nucleotides 530–542. Interestingly the deletion of this short region of 12 nucleotides dramatically impacted the binding affinity with NP (compare mutant 1 and mutant 3 in Table 2), suggesting this region is required for high affinity binding. This was further confirmed by the deletion of this short region in the wildtype UTR (Fig 8A, mutant 4), which significantly impacted its binding affinity with the NP (Fig 8D and Table 2). Taken together, Fig 8 demonstrates that the region from 530–542 nucleotides in the 5’ UTR of VCP mRNA is required for high affinity binding to the NP. This illustrates that 542^nd^ AUG is likely the integral part of NP binding site on the VCP mRNA 5’ UTR, which sheds light on the role of NP in boosting the mCherry expression in cells expressing bi-cistronic mRNA (Figs 5 and 6). Briefly, NP binding likely masks the 542^nd^ AUG in the VCP mRNA 5’UTR and shuts down the translation initiation from this start codon. However, NP associated ribosomes are internally loaded downstream of the 542^nd^ AUG, which finally engage at 988^th^ AUG and boost the mCherry expression in cells (Figs 5 and 6).

**Table 2:** Filter binding analysis to study the interaction of NP with the mutant sequences of the VCP mRNA 5’ UTR at different salt concentrations.

NaCl Concentration	80 mM	160 mM	320 mM
Interactors	K_d_ ±SD	K_d_ ±SD	K_d_ ±SD
NP + Mutant 1	42.2±10.5 nM	39.7±12.3, nM	33.9±8.2, nM
NP + Mutant 2	1.6±0.5, μM	1.9±1.4, μM	ND
NP + Mutant 3	2.4±0.6, μM	2.4±1.6, μM	ND
NP + Mutant 4	1.4±0.4, μM	3.9±2.3, μM	ND

Note: “ND” stands for not determined. Due to weak binding the binding profiles could not be completed and thus K_d_ values were not determined.

### VCP regulates the egress of hantavirus particles and other steps of virus replication cycle in HUVECs

**Fig 9:**
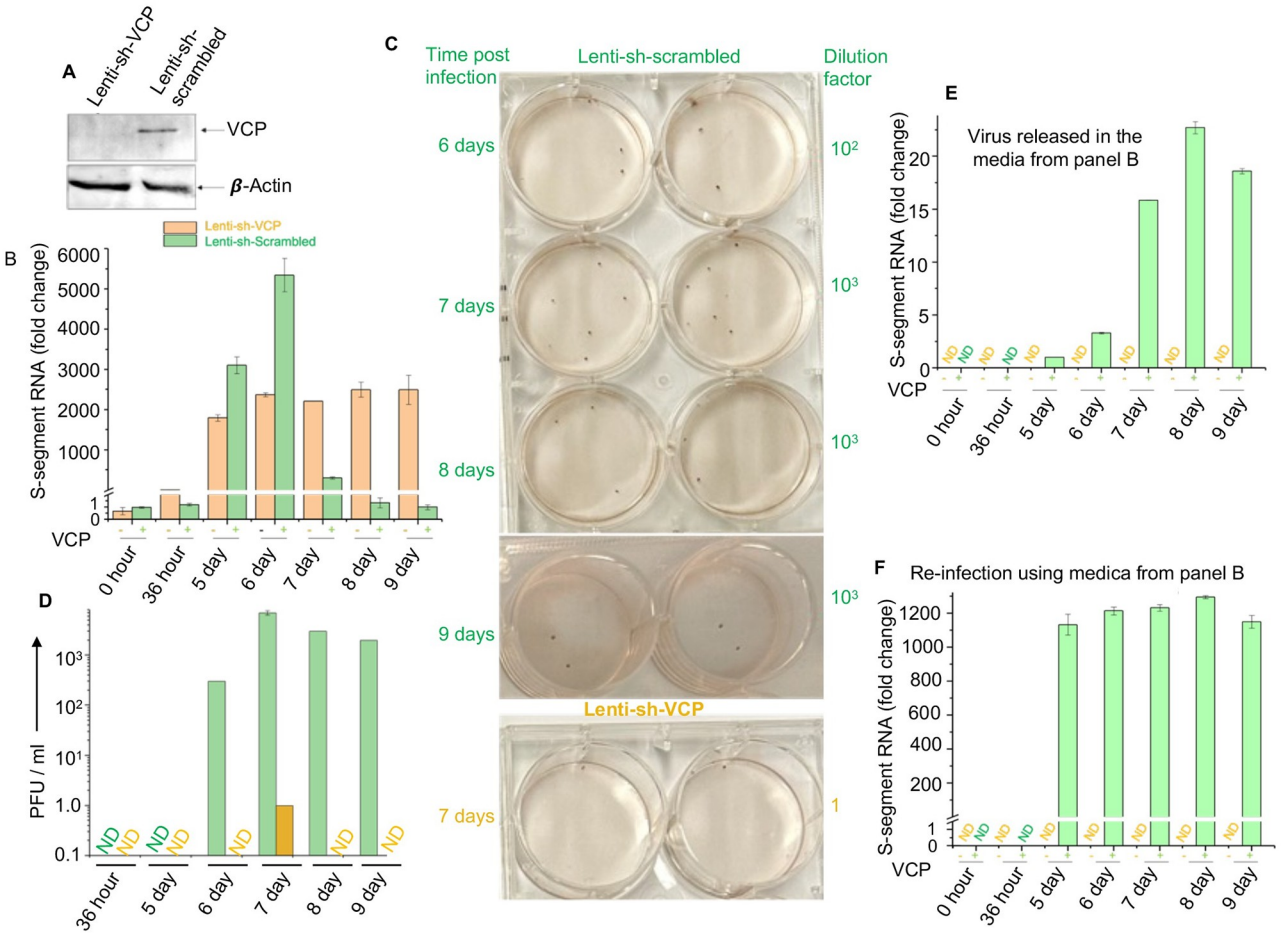
VCP regulates the egress of hantavirus particles from HUVECs. **(A)** Western blot showing the knockdown of VCP in HUVECs transduced with lentivirus expressing either scrambled shRNA or shRNA specific to VCP. (**B**) HUVECs transduced with lentivirus expressing either scrambled shRNA (brown) or VCP specific shRNA (green) were infected with PHV 24 hours post-transduction. Both cells and media were harvested at increasing time points post-PHV infection and viral S-segment RNA was quantified in harvested cells by real time PCR. (**C**) Viral titers in the harvested media were determined by the chemiluminescence plaque assay, as described in materials and methods. Media from each day post-infection was analyzed in duplicates as shown. (**D**) The foci from panel C were used to calculate PFU/ml, which was then plotted verses the corresponding day post-infection. (**E**) Quantification of viral S-segment RNA in the harvested media from panel B. The virus released in the media was concentrated by sucrose cushion before real time PCR was performed (see methods for details). (**F**) Using the data from panel E equal amount of virus released in the harvested media was added to fresh HUVECs, and cells were harvested 6 days post infection. Viral load was determined by real time PCR as mentioned above.

Since NP facilitates the translation of VCP in transfected and virus infected cells (Fig 2), probably by inhibiting the ribosome engagement at the 542^nd^ AUG in the 5’ UTR to promote the translation of downstream VCP ORF. It is thus critical to determine the role of VCP in hantavirus replication. The VCP gene was knocked down in HUVECs using the Lentivirus shRNA delivery system, as mentioned in materials and methods. Western blot analysis revealed a dramatic knockdown of VCP in 24 hours post lentivirus transduction (Fig 9A). To examine the impact of VCP knockdown on the hantavirus replication, HUVECs grown in six well plates were transduced with lentivirus, expressing either scrambled or VCP specific shRNA. Twenty-four hours post transduction, cells were infected with Prospect Hill virus (PHV) at an MOI of 0.1. Both media and virus infected cells from desired wells were harvested at increasing time points post infection. Viral load in cells was determined by the quantification of viral S-segment RNA using real time PCR (Fig 9B). Viral titers in the media were determined by the chemiluminescence based plaque assay (Fig 9C and 9D), as described in Materials and Methods. In addition, virus released in the media was concentrated by sucrose cushion and quantified by the estimation of S-segment RNA using real time PCR (Fig 9E). Viral load gradually increased up to six days post-infection in HUVECs transduced with lentivirus expressing scrambled shRNA (Fig 9B), followed by virus release in the media (green bars in Fig 9D and 9E). The real time PCR approach revealed very low virus release in the media on day 5 post-infection (Fig 9E), which remained undetected upon 100-fold dilution by the chemiluminescence plaque assay (Fig 9D).

Interestingly, viral load slowly increased in VCP knockdown cells up to 5 days post-infection and remained consistently high thereafter (Fig 9B brown bars) without any detectable virus release in the media, except on day 7 which showed negligible viral titers in the media (1PFU/ml) by the chemiluminescence plaque assay (brown bar in Fig 9D). However, the titers remained undetected by the real time PCR approach (Fig 9E). To further confirm that knockdown HUVECs didn’t support virus release, the harvested media from transduced cells was added to fresh wild type HUVECs and virus replication was examined by real time PCR 6 days post-infection (Fig 6F). Again, the media from transduced cells expressing scrambled shRNA generated high viral load in HUVECs. On the other hand, the media from VCP knockdown cells didn’t generate the detectable viral load in HUVECs. This experiment clearly demonstrates that although PHV slowly replicated in VCP knockdown HUVECs but viral egress in the media was inhibited.

**Fig 10:**
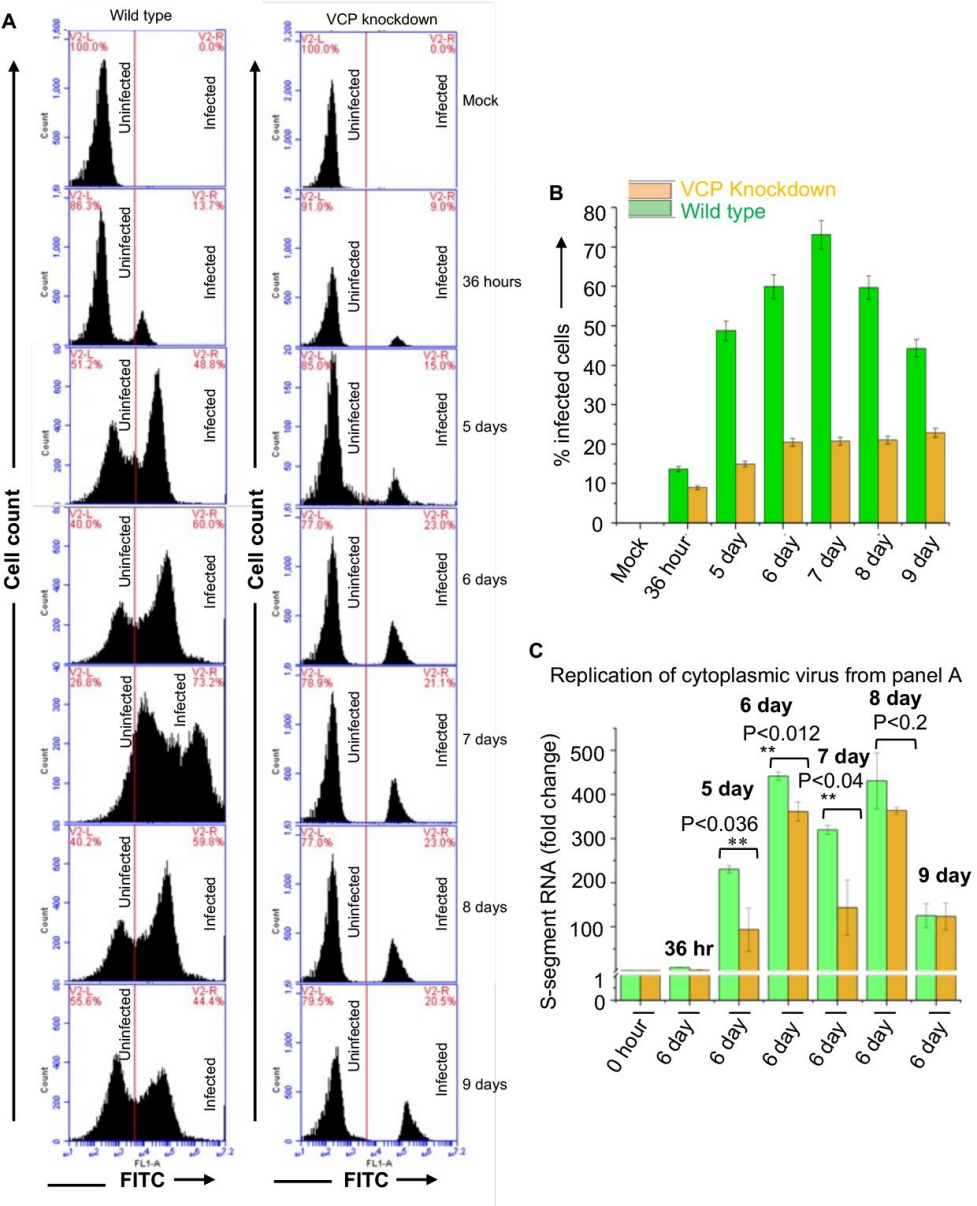
FACS analysis of PHV infected wild type and VCP knockdown HUVECs. (**A**) Wild type and VCP knockdown HUVECs were infected with PHV at an MOI of 0.1, Cells were harvested at increasing time points post infection and examined by FACS analysis using monoclonal ant-NP antibody and a secondary antibody conjugated with FITC, as described in materials and methods. (**B**) Number of percent infected cells from panel A were plotted verses the corresponding time point post post-infection. (**C**) Wild type and VCP knockdown HUVECs were infected with PHV as mentioned above. Infected cells were harvested at increasing time points post infection. The cytoplasmic virus obtained from wildtype (green bars) and VCP knockdown (Brown bars) harvested cells was used to re-infect fresh wild type HUVECs, followed by the examination of virus replication by real time PCR 6 days post-infection, as described in materials and methods.

Since VCP is involved in diverse cellular processes including protein degradation [16], protein trafficking through Golgi [17], Golgi-ER membrane fusion and Golgi assembly [11], it is possible that its knockdown may not only impact viral egress but also other steps of virus replication cycle. To gauge further mechanistic insight in the role of VCP on hantavirus replication, we examined viral dissemination to neighboring cells and quantified the replication efficiency of virus particles accumulated in the cytoplasm of VCP knockdown cells. Briefly, wild-type or VCP knockdown HUVECs were infected with PHV at an MOI of 0.1, as mentioned above. Cells were harvested at increasing time points post-infection, followed by the quantification of virus infected cells by the FACS analysis using anti-NP monoclonal primary antibody and secondary antibody conjugated with FITC, as mentioned in Materials and Methods. It is evident from Fig 10A and 10B that that virus dissemination gradually increased up to 7 days post-infection in wildtype HUVECs, followed by slight decrease likely due to viral egress. However, a marginal increase in the viral spread was observed in VCP knockdown HUVECs up to 6 days pos-infection without further spread up to 9 days post-infection. This demonstrates that VCP regulates the hantavirus dissemination to neighboring cells during the course of infection.

To examine the replication efficiency of virus particles accumulated in the cytoplasm, wild-type and VCP knockdown HUVECs were infected with PHV as mentioned above. Virus infected cells were harvested at increasing time points post-infection. Cells were disrupted by freez thaw cycles and virus in the cytoplasmic fractions was quantified by real time PCR. Based on vRNA equivalents, equal amount of cytoplasmic virus was used to infect fresh HUVECs and the viral load in the infected cells was determined by real time PCR 6 days post-infection. As shown in Fig 10C, the replication efficiency of virus accumulated in the cytoplasm of VCP knockdown cells was relatively low in comparison to wildtype cells (compare green and yellow bars). This suggests that VCP may play a role in other steps of hantavirus replication cycle.

### Chemical inhibition of VCP shuts down PHV replication in cells

**Fig 11:**
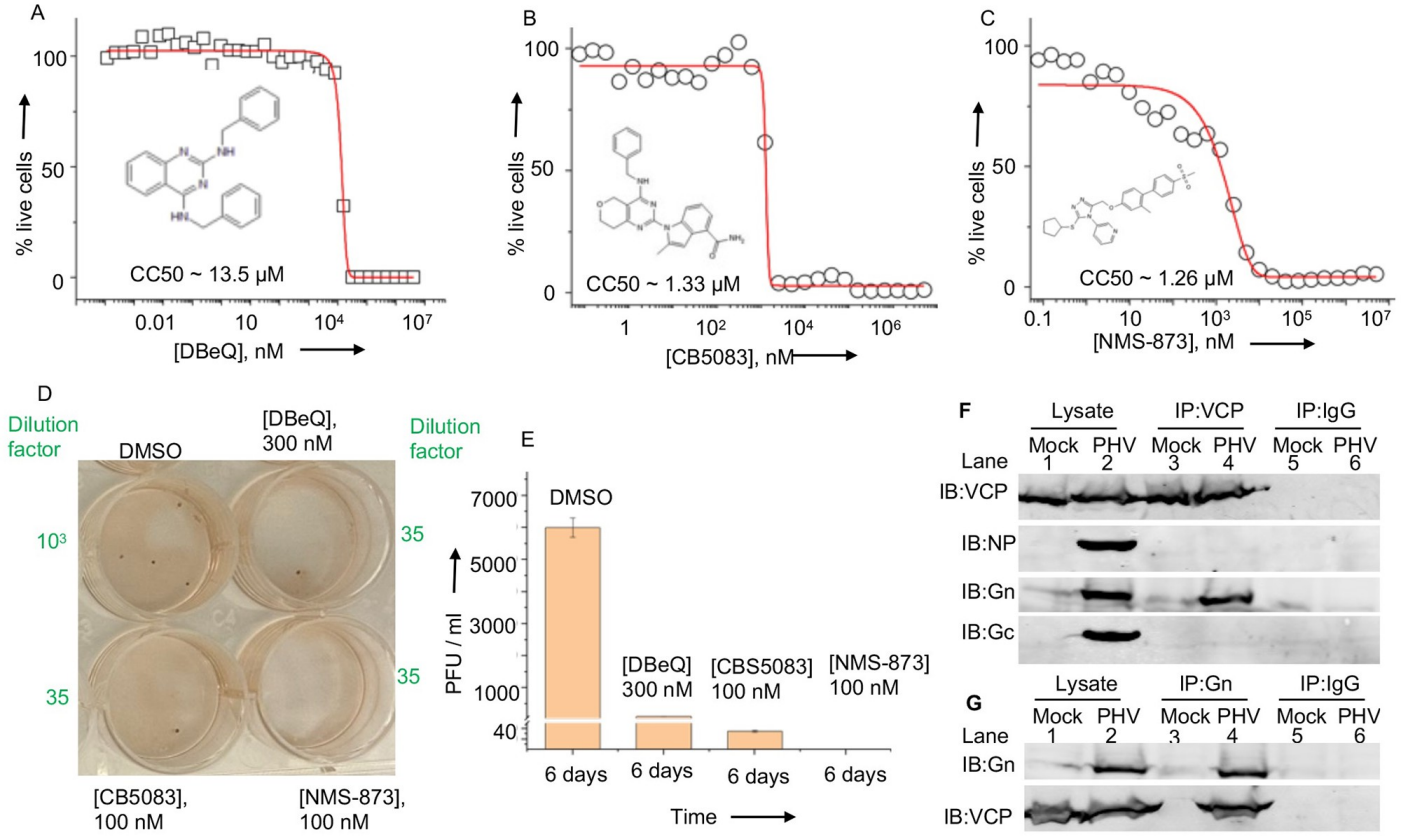
Chemical inhibition of VCP inhibits PHV replication in cells. Cytotoxicity of DBeQ (**A**), CB5083 (**B**) and NMS-873 (**C**) in HUVECs. The CC_50_ values are shown. (**D**) PHV infected HUVECs were incubated with either DBeQ (300 nM), or CB5083 (100 nM) or NMS-873 (100 nM) or with vehicle (DMSO) for 6 days post infection. Media from treated cells was harvested and examined by chemiluminescence plaque assay. Shown are the plaques developed in cells 6 days post-infection by the harvested media, using chemiluminescence plaque assay. (**E**) The foci in panel D were used to calculate the viral titers (PFU/ml) and plotted verses the corresponding treatment. (**F**) HUVECs either mock infected or infected with PHV were lysed and the resulting lysates were immunoprecipitated (IP) with either anti-VCP antibody or with IgG as control. The immunoprecipitated material was examined by western blot (IB) analysis using appropriate antibody, as shown. (**G**) The lysate from panel F was immunoprecipitated using anti-Gn antibody and the immunoprecipitated material was examined by western blot (IB) analysis using either anti-Gn or anti-VCP antibody, as shown.

Since VCP regulates the hantavirus replication cycle, especially the viral egress and dissemination to neighboring cells, we asked whether chemical inhibition of VCP inhibits the egress of PHV particles from infected HUVECs. As mentioned in Materials and Methods, we first used the CellTiter-Glo luminescence assay to examine the cytotoxicity of three chemical inhibitors of VCP (DBeQ, CB5083 and NMS-873) in HUVECs to ensure the potential viral inhibition is not due to cytotoxic effects of the chemical inhibitors. As shown by Fig 11A, 11B and 11C, the three chemical inhibitors DBeQ, CB5083 and NMS-873 were tolerated by HUVECs in μM range revealing the CC50 (the inhibitor concentration at which 50% cell death occurs) values of ~13.5 μM, ~1.33 μM and ~1.26 uM, respectively. DBeQ competitively inhibits the AAA ATPase activity of VCP by specifically binding to the ATPase site located in the VCP D2 domain [26]. DBeQ was later used in the optimization efforts that lead to the identification of CB5083 with improved VCP inhibition [27]. On the other hand, NMS-873 functions by binding to the interface of two adjacent domains within the active hexameric structure of VCP/p97 and leads to an interruption of its catalytic cycle by stabilizing the ADP-bound state [28]. To examine the antiviral efficacy, infected HUVECs were incubated with a single dose of DBeQ, CB5083 and NMS-873 at a concentration of 300 nM, 100 nM and 100 nM, respectively, for 6 days post-infection. Viral titers in the media were examined by the chemiluminescence plaque assay, as mentioned in Materials and Methods. It is evident from Fig 11D and 11E, that a single dose of DBeQ, CB5083 decreased the viral titers in the media by more than 110 fold. In comparison the similar treatment with NMS-873 decreased the viral titers to an undetectable level. This further confirms the critical role VCP in hantavirus replication and egress, and demonstrates that DBeQ, CB5083 and NMS-873 hold promise for therapeutic intervention of hantavirus disease.

### VCP binds to the hantavirus glycoprotein Gn

We next used immunoprecipitation and western blot analysis to determine whether VCP interacts with any viral components during the course of infection. Briefly, HUVECs were either mock infected or infected with PHV at an MOI of 0.1. Cells were harvested six days post infection and cell lysates were immunoprecipitated using either anti-VCP antibody or IgG as negative control. The immunoprecipitated material was examined by western blot analysis using either anti-NP, or anti-Gn or anti-Gc antibodies, as mentioned in materials and methods. It is evident from Fig 11F that Gn co-purified with VCP, suggesting a possible interaction between VCP and Gn. The co-purification of Gn alone suggests that VCP likely interacts with Gn unincorporated into assembled virus particles. The interaction was further confirmed by reverse immunoprecipitation using anti-Gn antibody for immunoprecipitation and anti-VCP antibody for western blot analysis (Fig 11G).

## Discussion

The hantavirus NP, encoded by the viral S-segment RNA is highly conserved among different hantavirus species. The sequence alignment of 34 different hantaviruses revealed a very high sequence homology. Some of the functional domains showing 100% conservation among different hantaviruses [29,30]. NP is the internal component of the virion structure, it suffers less selective pressure from the host immune response in comparison to the envelop glycoproteins, which likely justifies its high structural conservation [29,30]. NP is abundantly expressed in cellular cytoplasm during the course of virus infection. We previously reported that NP specifically binds to the RNA panhandle structure, formed by base pairing of complementary nucleotides at the 5’ and 3’ termini of the viral genome. The specific NP-panhandle interaction likely plays a role in selective encapsidation and packaging of the viral genome. We also reported that NP binds with high affinity to the conserved triplet repeat sequence UAGUAGUAG of the viral mRNA 5’ UTR. In addition, NP also binds to the ribosomal protein RPS19, a structural component of the 40S ribosomal subunit. The simultaneous binding to both the viral mRNA 5’ UTR and the 40S ribosomal subunit facilitates the engagement of NP associated ribosomes on viral mRNA. This selective ribosome loading on viral transcripts facilitates their translation. NP reserves a population of host cell ribosomes for the preferential translation of viral mRNA thereby avoiding their competition with host cell transcripts for the same host translation system.

Based on these previously reported findings we proposed that host cell transcripts harboring high affinity binding sites in their 5’ UTR sequences for hantavirus NP will also be preferentially translated by the NP-mediated translation strategy. To test this hypothesis HUVECs were transduced with lentiviruses expressing hantavirus NP. An examination of the transduced cell lysates by 2D-DIGE revealed an increase in the intrinsic steady-state levels of an array of host cell factors (Fig 1). An examination by STRING analysis demonstrated that majority of them are associated with the protein processing in the endoplasmic reticulum. The 2D-DIGE analysis also revealed a set of downregulated host cell factors, discussed later. An examination of both mRNA and protein levels of VCP in lentivirus transduced and hantavirus infected HUVECs confirmed that enhanced expression of VCP was likely due to the preferential translation of VCP mRNA by the NP mediated translational strategy and not due to transcriptional upregulation of the VCP mRNA (Fig 2). Previously reported transcriptomic analysis of HUVECs at increasing time intervals post infection by different hantavirus species, including PHV, revealed up and down regulation of numerous host cell genes at transcriptional level [31]. Interesting, majority of the regulated genes were involved in the host immune response [31]. This previously reported study did not show the transcriptional regulation of any of the host factors reported here (Fig 1). This suggests that upregulation of host factors (Fig 1) is likely due to preferential translation of their mRNAs by NP-mediated translation strategy.

To determine whether NP with the assistance of VCP mRNA 5’ UTR favors the translation of downstream ORF, the VCP 5’ UTR was fused with GFP ORF (Fig 3). The co-expression of resulting fusion transcript in NP expressing cells demonstrated that NP with the assistance of VCP mRNA 5’ UTR favored the translation of downstream GFP ORF. The VCP mRNA has six AUG codons in the 5’ UTR that have potential to generate short polypeptides (Fig 4A). The correct start codon for VCP is located 988 nucleotides from the 5’ cap (988^th^ AUG). Interestingly, the translation of synthetic fusion transcript in rabbit reticulocyte lysates demonstrated that translation initiation at the 542^nd^ AUG codon in the 5’UTR generated a 14KDa polypeptide. This translation initiation event negatively impacted the translation initiation at the correct start codon (988^th^ AUG) of the downstream GFP ORF. However, the addition of purified NP to the *in vitro* translation reaction inhibited the synthesis of 14KDa polypeptide and triggered the translation of downstream GFP ORF. Similarly, the elimination of 542^nd^ AUG by point mutation inhibited the synthesis of 14KDa polypeptide and facilitated the translation of GFP ORF (Fig 4). These *in vitro* studies demonstrated that apart from correct start codon (988^th^ AUG) the VCP mRNA harbors an additional start codon (542^nd^ AUG) that has strong potential to initiate translation. This observation became clearer when a dual reporter mRNA harboring GFP ORF downstream of the 542^nd^ AUG and mCherry ORF downstream of 988^th^ AUG, was translated in cells co-expressing NP (Figs 5 and 6). Interestingly, a strong GFP expression and negligible mCherry expression from the dual reporter transcript in transfected cells confirmed that translation initiation from the 542^nd^ AUG codon in the VCP mRNA 5’ UTR negatively regulates the translation of the ORF downstream of the 988^th^ AUG. However, the co-expression of NP suppressed the GFP expression and triggered the mCherry expression from the dual reporter mRNA in cells. Further studies revealed that NP binds to the VCP mRNA 5’UTR *in vitro* with high affinity and the NP binding site is likely located in the region from 530–542 nucleotides (Figs 7 and 8). Taken together these interesting observations suggest that high affinity binding of NP to the VCP mRNA 5’ UTR likely masks the 542^nd^ AUG and thus inhibit the ribosome engagement at this start codon, preventing the synthesis of 14KDa polypeptide. Since NP is known to bind the 40S ribosomal subunit, It is possible that NP associated ribosomes are loaded on the VCP mRNA 5’UTR downstream of the 542^nd^ AUG that ultimately engage at the 988^th^ AUG codon to initiate the translation of the downstream ORF. However, if NP binding site is located within the ORF of an mRNA molecule, the NP associated ribosomes will not be loaded at the correct initiation codon, which might trigger the degradation of the transcript by the host RNA degradation machineries. It is probable that such an incorrect ribosome loading on transcripts in NP expressing cells trigged their degradation, resulting in the reduction of their translation products in cells, as observed by 2D-DIGE analysis (Fig 1).

Although translation initiation at 542^nd^ AUG in the VCP mRNA 5’UTR is evident from *in vitro* and cell culture studies, it is still unclear whether the 14KDa polypeptide is produced from the full length VCP mRNA in cells. Further studies, especially the generation of antibodies against the 14KDa polypeptide, will be required to determine its expression, stability, and potential role in the biology of VCP.

The knockdown experiment unveiled a compelling function of VCP in hantavirus replication. Although the knockdown of VCP in HUVECs slowed down virus replication during the early stages of virus replication, the viral load in the cellular cytoplasm remained consistently high (Fig 9). Examination of cell culture media revealed that viral egress in knockdown cells was inhibited, justifying the consistently elevated viral load in the cytoplasm of VCP knockdown HUVECs. It became imperative to ascertain whether the virus particles accumulating in the cellular cytoplasm due to VCP knockdown retained their infectivity. Given VCP’s involvement in various cellular processes, including Golgi-ER membrane fusion and Golgi assembly [11], we posited that VCP might influence other stages of the hantavirus replication cycle. This speculation gained support from observations demonstrating that virus particles accumulating in the cytoplasm of VCP knockdown cells exhibited poor replication efficiency when used to re-infect fresh HUVECs (Fig 10C). Moreover, VCP knockdown impeded viral spread to neighboring cells during the course of infection (Fig 10A and 10B). These findings indicate that VCP might play a role at multiple stages of the hantavirus replication cycle. The involvement of VCP in the hantavirus replication cycle, particularly in viral egress, was substantiated by chemically inhibiting VCP using three well-characterized inhibitors (DBeQ, CB5083, and NMS-873). All three inhibitors effectively restrained hantavirus replication in cells (Fig 11).

The role of VCP in the endocytic trafficking of classical swine fever virus has been documented previously [32]. Reports on VCP’s interaction with HIV-1 gp41 and gp60 in the endoplasmic reticulum and Golgi complex underscore its significance in the HIV life cycle [33,34]. Intriguingly, immunoprecipitation studies revealed VCP’s binding to the hantavirus glycoprotein Gn before its incorporation into assembled virions. It is plausible that VCP is involved in transporting Gn to the site of virus assembly on Golgi-ER membranes. However, the actual mechanism by which the VCP-Gn interaction facilitates the production of infectious hantavirus particles and promotes their egress from the host cell remains elusive.

## Materials and methods

### Plasmids

Straightforward cloning techniques were used to generate the constructs used in this study. Briefly, the DNA segment encoding the 5’ UTR of VCP mRNA was synthesized by GenScript. The synthetic DNA segment was fused to GFP ORF upstream of the start codon by PCR. The resulting PCR product was cloned in pCDNA 3.1 (+) backbone to generate the plasmid P1 (Fig 3), as previously reported [35]. Same strategy was used for the construction of plasmid P4, expressing the GFP mRNA harboring the randomized sequence of the 5’UTR of VCP mRNA. The plasmid P2 (Fig 3) has an IRES sequence upstream of mCherry ORF and is expressed under CMV promoter. This plasmid was a gift from Dr. Que (Kansas University Medical center). The plasmid P3 (Fig 3) was constructed by the incorporation of Sin Nombre virus NP ORF upstream of the IRES sequence in the plasmid P2. The mRNA expressed from plasmid P3 under CMV promoter is translated by both cap dependent and IRES mediated translation strategies, generating NP and mCherry, respectively. The dual reporter plasmid (pGFP-mCherry) was constructed by Genscript. The DNA segment encoding the dual reporter mRNA (Figs 5 and 6) was synthesized and cloned in pCDNA3.1(+) backbone to generate the pGFP-mCherry construct. The plasmids pLenti-GFP and pLenti-NP were generated by cloning the open reading frames of GFP and Sin Nombre virus or Prospect Hill virus NP into the pLenti-CMV vector, as previously reported [21]. The packaging plasmid psPAX2 and the envelope plasmid pMD2.G were from Addgene. The plasmid pLKO.1—scramble shRNA was a gift from David Sabatini (Addgene plasmid # 1864) [36]. The pLKO.1-VCP shRNAs were constructed by inserting the hairpin sequences into pLKO.1—TRC cloning vector, a gift from David Root (Addgene # 10878) [37]. The target sequences for shRNA knockdown of VCP were: 5’—AGGGAGGTAGATATTGGAATT-3’ and 5’—GATGGATGAATTGCAGTTGTT-3’. Alternatively, Lenti-X Packaging Single Shots (VSV-G) (Takara Cat# 631276) was used for lentivirus production, following the manufacturer’s instructions. The lentivirus particles were concentrated using Lenti-X Concentrator (TakaRa Cat# 631231) following the manufacturer’s instructions.

### Cell culture, virus propagation and lentivirus preparation

Human embryonic kidney 293T (HEK293T), African green monkey kidney (Vero E6) and Baby hamster kidney cells (BHK21) were grown in DMEM (HyClone) supplemented with 10% fetal bovine serum (HyClone), 2 mM L-glutamine, 100 U/ml penicillin and 100 μg/ml streptomycin. The media for BHK21 cells also contained 5% tryptose phosphate broth (Gibco). Human umbilical vein endothelial cells (HUVEC) were purchased from Lonza and cultured in EGM BulletKit medium (Lonza). Alternatively, vascular cell base medium (ATCC, cat# PCS-100-030) along with endothelial cell growth kit-VEGF (ATCC, cat# PCS-100-041) was used to culture HUVECs. Lentiviruses were packaged in HEK293T cells. The expression plasmid pLenti-GFP or pLenti-NP was co-transfected into HEK293T cells along with packaging plasmid psPAX2 (Addgene) and envelop plasmid pMD2.G (Addgene). Supernatants from transfected cells were harvested 72 hours post-transfection. Lentiviral particles were concentrated by ultracentrifugation and quantified by qPCR assay [38]. Lentivirus particles for the knockdown of VCP gene were generated using the similar approach, except the pLenti-GFP or pLenti-NP plasmids were replaced by pLKO.1-VCP shRNA constructs. The VCP knockdown stable HUVEC cell lines were generated by puromycin selection (3 μg/ml), as previously reported [21]. Prospect Hill hantavirus (PHV) strain (MP40) and hazara virus (HZV) were obtained from World Reference Center for Emerging viruses and Arbaviruses-WRCEVA, UTMB Galveston. PHV was propagated in Vero E6 cells. Briefly, PHV was inoculated in Vero E6 cells at an MOI of 0.03. The cells were cultured in DMEM containing 2.5% FBS. Supernatant from cultured cells was collected thirteen days post-infection. HZV was propagated by inoculating BL21 cells at an MOI of 0.01. The cells were cultured in DMEM (HyClone) supplemented with 2% FBS (HyClone), 2 mM L-glutamine, 100 U/ml penicillin, 100 μg/ml streptomycin and 5% tryptose phosphate broth (Gibco). Supernatant from cultured cells was collected ten days post-infection. The viral titers in the supernatant were determined by plaque assay [39].

### Two-dimensional difference gel electrophoresis (2D-DIGE)

Lentiviruses expressing either Hantavirus NP or GFP as negative control were generated as mentioned above. HUVEC cells grown in 10 cm dishes were infected with the lentiviruses and cells were lysed 48 hours post-infection in RIPA lysis buffer (Fisher Scientific). Cell lysates were examined to determine the impact of NP expression on the translation of host cell mRNAs, using 2D-DIGE [40]. The 2D-DIGE and Protein ID by mass spectrometry was performed by Applied Biomics, Inc, Hayward, CA, as described below.

#### Preparation of samples

Protein lysate was exchanged into 2D cell lysis buffer. Protein concentration was measured using Bio-Rad protein assay method. Internal standard was made by mixing equal amount of protein from each sample.

#### CyDye labeling

For each sample, 30 μg of protein was mixed with 0.5 μl of diluted CyDye and kept in dark on ice for 30 min. GFP and NP samples were labeled with Cy3 and Cy5, respectively. The labeling reaction was stopped by adding 1.0 μl of 10 mM Lysine to each sample, and incubating in dark on ice for additional 15 min. The labeled samples were then mixed together. The 2X 2D Sample buffer (8 M urea, 4% CHAPS, 2% pharmalytes and trace amount of bromophenol blue), 100 μl destreak solution and Rehydration buffer (7 M urea, 2 M thiourea, 4% CHAPS, 1% pharmalytes and trace amount of bromophenol blue) were added to the labeling mix to make the total volume of 250 μl for the 13 cm IPG strip. The labeled samples were mixed well and spun down before loading into strip holder.

#### IEF and SDS-PAGE

After loading the labeled samples, IEF (pH 3–10 Linear) was run following the protocol provided by GE Healthcare. Upon finishing the IEF, the IPG strips were incubated in the freshly made equilibration buffer-1 (50 mM Tris-HCl, pH 8.8, containing 6 M urea, 30% glycerol, 2% SDS and trace amount of bromophenol blue) for 15 minutes with gentle shaking. Then the strips were rinsed in the freshly made equilibration buffer-2 (50 mM Tris-HCl, pH 8.8, containing 6 M urea, 30% glycerol, 2% SDS and trace amount of bromophenol blue) for 10 minutes with gentle shaking. Next the IPG strips were rinsed in the SDS-gel running buffer before transferring into 10.5% SDS-gels. The SDS-gels were run at 15 °C until the dye front ran out of the gels.

#### Image scan and data analysis

Gel images were scanned immediately following the SDS-PAGE using Typhoon TRIO (GE Healthcare). The scanned images were then analyzed by Image Quant software (version 6.0, GE Healthcare), followed by in-gel analysis using DeCyder software (version 6.5, GE Healthcare). The fold change of the protein expression levels was obtained from DeCyder analysis.

### Protein identification by mass spectrometry

Protein spots in the gel were picked and proteins identification was carried out using mass spectrometry [41], as discussed below.

#### Spot picking and Trypsin digestion

The spots of interest were picked up by Ettan Spot Picker (Amersham BioSciences) based on the in-gel analysis and spot picking design by DeCyder software. The gel spots were washed a few times then digested in-gel with modified porcine trypsin protease (Trypsin Gold, Promega). The digested tryptic peptides were desalted by Zip-tip C18 (Millipore). Peptides were eluted from the Zip-tip with 0.5 μl of matrix solution (α-cyano-4-hydroxycinnamic acid (5 mg/ml) in 50% acetonitrile, 0.1% trifluoroacetic acid, 25 mM ammonium bicarbonate) and spotted on the AB SCIEX MALDI plate (Opti- 384 Well Insert).

#### Mass spectrometry

MALDI-TOF MS and TOF/TOF tandem MS/MS were performed on an AB SCIEX 5800 System (AB SCIEX, Framingham, MA). MALDI-TOF mass spectra were acquired in reflectron positive ion mode, averaging 4000 laser shots per spectrum. TOF/TOF tandem MS fragmentation spectra were acquired for each sample, averaging 4000 laser shots per fragmentation spectrum on each of the 10 most abundant ions present in each sample (excluding trypsin autolytic peptides and other known background ions).

#### Database search

Both of the resulting peptide mass and the associated fragmentation spectra were submitted to GPS Explorer workstation equipped with MASCOT search engine (Matrix science) to search the database of National Center for Biotechnology Information non-redundant (NCBInr) or Swiss-Port database. Searches were performed without constraining protein molecular weight or isoelectric point, with variable carbamidomethylation of cysteine and oxidation of methionine residues, and with one missed cleavage also allowed in the search parameters. Candidates with either protein score C.I.% or Ion C.I.% greater than 95 were considered significant.

### Western blot and antibodies

Cells were washed once with phosphate buffered saline (PBS) and lysed with radioimmunoprecipitation assay buffer (RIPA buffer) supplemented with protease and phosphatase inhibitor cocktails (Roche). Clarified cell extracts were mixed with equal volume of 2× SDS loading buffer and boiled at 95°C for 5 min. Proteins were separated by SDS-PAGE and transferred to PVDF membrane (Millipore). The membrane was blocked with 5% non-fat milk in PBST buffer (1x PBS, 0.05% Tween 20) followed by incubating with primary and secondary antibodies diluted in blocking buffer. The secondary antibodies, donkey anti-rabbit (cat# 926803), goat anti-rat (cat# 9256807), donkey anti-mouse (Cat# 9266807) were from LI-COR. The blots were scanned using the Odyssey CLx imaging system from LI-COR. The primary antibodies for VCP (2648s) and GFP (2956s) were from Cell Signaling Technologies. The primary antibodies for Gn (Cat#7681) and Gc (Cat#7683) were from ProSci Inc. The polyclonal antibodies for HZV and PHV NP were produced in our lab, as previously reported [21,22]. The monoclonal anti-NP (hantavirus) antibody was from Invitrogen (Cat#: MA518208).

### Coimmunoprecipitation

Coimmunoprecipitation was carried out as previously described [21]. Briefly, HUVECs were infected with PHV at an MOI of 0.1 and harvested six days post-infection. Cells were lysed with NP-40 lysis buffer (50 mM Tris-HCl pH 7.5, 150 mM NaCl, 0.5% NP-40, 10% glycerol, 1 mM EDTA), supplemented with protease and phosphatase inhibitor cocktails (Roche). Ten percent of the clarified cell lysates were saved as input. The remaining cell lysates were incubated with 1 μg of required antibody for 4 hours at 4°C with gentle rotation. The antigen-antibody complexes were captured with 40 μl of protein G agarose beads (50% slurry) by continuous rotation for one hour at 4°C. The beads were washed four times with lysis buffer, resuspended in 1× SDS loading buffer and boiled at 95°C for 5 min. After brief centrifugation the supernatants were loaded into SDS PAGE gel, followed by western blot analysis using appropriate antibody.

### Chemiluminescence plaque assay

An established chemiluminescent plaque assay [42] was used to determine titers in the media harvested from PHV infected cells. Briefly, HUVECs or Vero E6 cells were seeded on gelatin coated 6 well plates one day prior to infection. Gelatin coated plates were prepared by the addition of 1 ml of 0.1% gelatin solution in water to each well of the six well plates, followed by incubation at room temperature for one hour. The gelatin was aspired just before seeding the cells. One ml dilution series of the test media containing the virus was generated using DMEM without antibiotics and serum. The resulting solutions were used to infect the cells grown on gelatin coated plates. Briefly, cells were washed thrice with 1x PBS, followed by the addition of one ml of diluted test media. The plates were incubated at 37 °C inside the CO_2_ incubator for one hour with brief swirling every 15 minutes. The media was aspired and cells were washed once with 1x PBS, followed by the addition of 3 ml overlay media. The overlay media was prepared by warming up the solution A (2x DMEM, 10% FBS, 2.5% Hepes +2x Pen/Strep) up to 50 °C in a water bath and preparing solution B (1% agarose in water). The solution B was heated to ensure the agarose is properly dissolved, followed by maintaining the temperature at 50 °C. The solutions A and B were mixed in 1:1 ratio to generate the overlay media which was immediately added to each well of the six well plate. The plate was incubated at 37 °C inside the CO_2_ incubator for 6 days. The Agarose block was removed from each well by injecting 2–3 ml of wash buffer (1x PBS supplemented with 0.15% Tween 20) under the agarose layer and turning the plate upside down. Cells were washed gently twice with wash buffer and fixed by adding 2ml of 100% methanol to each well, followed by incubation at -20°C for 10 minutes. Methanol was removed and wells were allowed to dry, followed by washing twice with wash buffer. 300 μl of the anti-NP monoclonal antibody (Invitrogen, Cat#: MA518208) at a dilution of 1:50 in antibody dilution buffer (wash buffer containing 5% FBS), were added to each well, followed by incubation at 37 °C for 1 hour in a humidified 5% CO_2_ incubator. The wells were washed five times with wash buffer, followed by the addition of 1 ml/well of goat anti-mouse secondary antibody conjugated with horseradish peroxidase (Genscript, Cat#: A00160) at a dilution of 1:1000 in antibody dilution buffer. Plates were incubated for one hour at 37°C in a humidified 5% CO2 incubator, followed by washing the cells five times with wash buffer. One ml of DAB solution was added to each well and incubated at room temperature until foci were detected. The DAB solution was prepared by dissolving 48 mg of DAB in 20 mL of 0.05 M Tris buffer (pH 7.6), followed by the addition of 0.7 ml of 3% H_2_O_2_. After the foci are detected, the reaction was terminated by washing the wells with water or 1x PBS.

### FACS analysis

FACS analysis was used to quantify the PHV infected cells at increasing time points post-infection using a standardized protocol [43]. Briefly, wild type or VCP knockdown cells in six well plates were infected with PHV at an MOI of 0.1. Cells were harvested at increasing time points post-infection. Cell pallets from each well were washed twice with 1x PBS and incubated with 5ml of 1x PBS containing 4% paraformaldehyde at room temperature for 30 minutes with gentle rocking. Cells were pelleted down, washed twice with 1x PBS and stored in 300 ul of 1x PBS at 4°C. The procedure was repeated up to 9 days pos-infection until all the cells were harvested, fixed, and stored at 4°C. On the day of FACS analysis cell pallets were briefly centrifuged and 1x PBS was removed, the resulting pellets were suspended in 500 μl of 1x PBS containing 0.05% Triton X-100, followed by incubation at room temperature for 30 minutes. Cells were pelleted down and washed twice with 1x PBS and incubated at room temperature for 1 hour with 300 μl of anti-NP monoclonal antibody (Invitrogen, cat#: MA5 18208) at a dilution of 1:2000 in 1x PBS. Cells were pelleted down, washed with 1x PBS and incubated for 30 minutes at room temperature with 300 μl of goat anti-mouse FITC conjugated secondary antibody (Sigma, Cat#: F0257) at a dilution of 1:32 in 1x PBS. This step was carried out in dark. Cells were pelleted down and washed twice with 1xPBS and finally suspend in 300 μl of 1x PBS for FACs analysis using BD C6 Plus instrument.

### Replication of cytoplasmic virus

Wild type and VCP knockdown HUVECs were infected in six well plates with PHV at an MOI of 0.1. Cells were harvested at increasing time points post infection, followed by disruption by freez thaw cycle. Briefly, harvested cells from each well were suspended in 300 μl of complete DMEM, frozen in dry ice and thawed at 37 °C water bath, followed by a brief vortex. This step was repeated 3–5 times. Cells were sonicated thrice for 30 seconds in ice with a 30 second rest on ice in between. Lysates were pelleted to remove the cell debris and the supernatant containing the PHV was saved. PHV was quantified in the supernatant by the estimation of S-segment RNA by real time PCR before using the supernatant for reinfection to fresh cells.

### Cytotoxicity

The impact of VCP inhibitors (DBeQ, CB5083 and NMS-873) upon the viability of HUVECs was determined using Cell Titer-Glo 2.0 luminescent assay reagent according to the manufacturer’s instructions (Promega, Cat # G9241), as previously reported [44]. Briefly, ~ 10,000 HUVECs were seeded in each well of a 96-well plate and incubated for 48 hours in 100 μl media containing increasing concentrations of the inhibitor. Control wells containing media with increasing concentrations of inhibitor without cells were also prepared. The plate was equilibrated at room temperature for 30 minutes, followed by the addition of 100 μl of the Cell titer-Glo reagent. The plate was incubated for 2 minutes on an orbital shaker at room temperature to induce cell lysis. The plate was incubated for additional 10 minutes at room temperature to stabilize the luminescent signal. The luminescence was recorded on a plate reader (PROMEGA GloMax Explorer). The luminescence signal for each sample was subtracted from the corresponding negative control. Since VCP inhibitors were dissolved in 1% DMSO, the cell viability in each well was normalized relative to viability observed at 1% DMSO without the inhibitor. The inhibitors DBeQ (Cat # 501873396), CB508 (Cat # 501365653)) and NMS-873 (Cat # NC1147220) were from Fisher.

### Purification of hantavirus wild type NP and NP mutant lacking the RNA binding domain

Expression of wild type SNVN NP of NP mutant lacking the RNA binding domain was carried out, as previously reported [44]. Briefly, BL21 (DE3) cells transformed with pTriEx1.1 vector harboring the NP gene, followed by induction with 1 mm isopropyl 1-thio-β-d-galactopyranoside upon entering into exponential growth phase (*A*_600_ = 0.4). Cells were allowed to grow for another 4 h at 37 °C and were harvested by centrifugation at 3000 rpm for 30 min at room temperature. Cells were resuspended in lysis buffer (20 mm HEPES, pH 8.0, 300 mm NaCl, 2 mm CHAPS, 8 m urea, 10 mm imidazole, and protease inhibitors (protease inhibitor mixture, Thermo Scientific). Cleared lysates were loaded on the 5ml HisTrap column (17-5286-01, GE healthcare) and purification was carried out on AKTA pure protein purification system (GE Healthcare) as previously reported [45]. The column was washed three times with lysis buffer containing increasing concentrations of 25 mM, 50 mM and 100 mm imidazole. The bound protein was finally eluted with lysis using an imidazole gradient from 0–250 mM. Purified protein was refolded by step dialysis in 20 mm HEPES, pH 8.0, 200 mm NaCl, 5% glycerol, and 1 mm DTT with a gradual decrease in the concentration of urea.

### Real time PCR

Total RNA from virus infected or control cells was purified using RNeasy Kit (Qiagen) and reverse transcribed using M-MLV Reverse Transcriptase (invitrogen) according to manufacturer’s instructions. Real time PCR reactions were performed on ABI 7500 real time PCR system (Applied Biosystems), using SYBR green PCR master mix (Roche), as previously reported [22]. The mRNA levels of a housekeeping gene β-actin were quantified as an internal control. The relative quantification method was used for data analysis as previously reported [21]. The primers used for the quantification of β-actin mRNA and hantavirus S-segment RNA have been reported previously [21].

### T7 Transcription for the synthesis of wild type and mutants of VCP mRNA 5’ UTR

The DNA sequences encoding the VCP mRNA 5’ UTR and random 5’ UTR were PCR amplified from plasmid P1 and plasmid P4, respectively, using the appropriate forward and reverse primer. The forward primers contained a flanking T7 promoter at the 5’ terminus. As previously reported [46], overlapping PCR was used to incorporate a point mutation in the VCP mRNA 5’ UTR at 545 nucleotides from the 5’ terminus by converting the AUG codon to AUC. The DNA segments encoding other deletion mutants used in Fig 8 were generated using similar approach. The PCR products were gel purified and used as template in an *in vitro* T7 transcription reaction. RNA synthesis was carried out using the T7 RiboMax kit (Promega), following the manufacturer’s instructions. The RNA was either biotinylated or radiolabeled with [α^32^P] GTP during synthesis as previously reported [35,47,48].

### RNA filter binding analysis

Interaction of bacterially expressed and purified NP with the radiolabeled synthetic UTR sequences (Fig 7, Table 1) was studied by filter binding assay, as previously reported [10,48]. All binding reactions were carried out in RNA binding buffer (40 mM Tris-HCl [pH 7.4], 80 mM NaCl, 20 mM KCl, 1 mM dithiothreitol) at a fixed concentration of synthetic 5’UTR with increasing concentrations of NP. Reaction mixtures were incubated at room temperature for 30–45 min and filtered through nitrocellulose membranes under vacuum. Filters were washed with 10 ml of RNA binding buffer and dried. The amount of radiolabeled RNA retained on the filter at each input concentration of NP was measured by quantifying the radioactive signal, using a scintillation counter. The background signal from nonspecific binding of RNA to the filter was subtracted from each data point. A binding profile was generated by plotting the radioactive signal along Y-axis and NP concentration along X-axis. The percentage of bound RNA at each input concentration of NP was calculated using the Eq 1.

$$\text{Percentage}\;\text{of}\;\text{bound}\;\text{RNA}=\Delta R/\Delta R_{\text{max}}*100$$ (1)

Where ΔR is the change in radioactive signal at each addition of NP. ΔR_max_ is the same parameter when the RNA is totally bound to the NP. Double reciprocal plot (1/*ΔR* versus 1*/C*_*p*,_ [10]) was used to calculate the value of *ΔR*_*max*_, using Eq 2. C_p_ is the input NP concentration.


$$1/\Delta R=1/\Delta R_{max}+K_{d}/(\Delta R_{max}*C_{p}).$$ (2)

Since Eq 2 is valid for the calculation of *ΔR*_*max*_ under the conditions where Cp >> initial concentration of RNA [49], only the data points corresponding to the saturation phase of the binding profile were fitted to the Eq 2 for the calculation of *ΔR*_*max*_. Alternatively, the *ΔR*_*max*_ was calculated by simply averaging the radioactive signal of saturating data points. The percentage of bound RNA obtained from Eq 1 was plotted verses input NP concentration, and the resulting data points were fit to one site specific binding model using the program Origin 6 (Microcal). The apparent dissociation constant (*Kd*) corresponded to the concentration of NP required to obtain the half-saturation in the fitted binding curve, assuming that the complex formation obeys a simple bimolecular equilibrium.

### Biolayer interferometry (BLI)

Biolayer interferometry was used to examine the binding affinity of purified NP with the synthetic biotinylated 5’UTR sequence of VCP mRNA, using the BLITZ system (ForteBio Inc.), as previously reported [35,47,48]. Briefly, the synthetic biotin-5’ UTR was loaded onto a high precision streptavidin biosensors (catalog # 18–5019, Forte Bio Inc.), as previously reported [46]. All reactions were carried out at room temperature in RNA binding buffer (20 mM Tris-HCl, pH 7.4, 80 mM NaCl, 20mM KCl, and 1mM DTT). After mounting the RNA, the biosensors were equilibrated in RNA binding buffer and then dipped in the purified NP solution for the measurement of association kinetics. The reaction cycles were as follows: initial base line for 30 seconds, loading of biotinylated RNA on streptavidin biosensors for 150 seconds, base line for 30 seconds, association of protein with the RNA for 120 seconds, followed by dissociation phase of 120 seconds. The kinetic parameters K_ass_ (association rate constant), K_dis_ (dissociation rate constant) and the binding affinities (K_d_ = K_dis_/K_ass_) were calculated with the help of inbuilt data analysis software (BLItZ Pro), as previously reported [35,47,48].
